# *Kingella kingae* Expresses Four Structurally Distinct Polysaccharide Capsules That Differ in Their Correlation with Invasive Disease

**DOI:** 10.1371/journal.ppat.1005944

**Published:** 2016-10-19

**Authors:** Kimberly F. Starr, Eric A. Porsch, Patrick C. Seed, Christian Heiss, Radnaa Naran, L. Scott Forsberg, Uri Amit, Pablo Yagupsky, Parastoo Azadi, Joseph W. St. Geme

**Affiliations:** 1 Department of Pediatrics and Department of Molecular Genetics and Microbiology, Duke University Medical Center, Durham, NC; 2 Department of Pediatrics, The Children's Hospital of Philadelphia, Philadelphia, PA; 3 Complex Carbohydrate Research Center, University of Georgia, Athens, GA; 4 Radiation Oncology, Chaim Sheba Medical Center, Ramat Gan, Israel; 5 Soroka University Medical Center, Ben-Gurion University of the Negev, Beer-Sheva, Israel; 6 University of Pennsylvania Perelman School of Medicine, Philadelphia, PA; University of Birmingham, UNITED KINGDOM

## Abstract

*Kingella kingae* is an encapsulated gram-negative organism that is a common cause of osteoarticular infections in young children. In earlier work, we identified a glycosyltransferase gene called *csaA* that is necessary for synthesis of the [3)-β-Gal*p*NAc-(1→5)-β-Kdo*p*-(2→] polysaccharide capsule (type a) in *K*. *kingae* strain 269–492. In the current study, we analyzed a large collection of invasive and carrier isolates from Israel and found that *csaA* was present in only 47% of the isolates. Further examination of this collection using primers based on the sequence that flanks *csaA* revealed three additional gene clusters (designated the *csb*, *csc*, and *csd* loci), all encoding predicted glycosyltransferases. The *csb* locus contains the *csbA*, *csbB*, and *csbC* genes and is associated with a capsule that is a polymer of [6)-α-Glc*p*NAc-(1→5)-β-(8-OAc)Kdo*p*-(2→] (type b). The *csc* locus contains the *cscA*, *cscB*, and *cscC* genes and is associated with a capsule that is a polymer of [3)-β-Rib*f*-(1→2)-β-Rib*f*-(1→2)-β-Rib*f*-(1→4)-β-Kdo*p*-(2→] (type c). The *csd* locus contains the *csdA*, *csdB*, and *csdC* genes and is associated with a capsule that is a polymer of [P-(O→3)[β-Gal*p*-(1→4)]-β-Glc*p*NAc-(1→3)-α-Glc*p*NAc-1-] (type d). Introduction of the *csa*, *csb*, *csc*, and *csd* loci into strain KK01Δ*csa*, a strain 269–492 derivative that lacks the native *csaA* gene, was sufficient to produce the type a capsule, type b capsule, type c capsule, and type d capsule, respectively, indicating that these loci are solely responsible for determining capsule type in *K*. *kingae*. Further analysis demonstrated that 96% of the invasive isolates express either the type a or type b capsule and that a disproportionate percentage of carrier isolates express the type c or type d capsule. These results establish that there are at least four structurally distinct *K*. *kingae* capsule types and suggest that capsule type plays an important role in promoting *K*. *kingae* invasive disease.

## Author Summary

*Kingella kingae* is a gram-negative pathogen that is being recognized increasingly as a cause of joint, bone, and other bloodborne infections in young children, reflecting advances in cultivation techniques and molecular methods of detection. Previous work established that *K*. *kingae* expresses a polysaccharide capsule, a surface factor that likely plays a key role in allowing the organism to transition from colonization of the oropharynx to survival in the bloodstream. We analyzed a large collection of epidemiologically diverse *K*. *kingae* isolates and found that there are at least four structurally distinct capsule types in the *K*. *kingae* population. In addition, we found that two of the four capsule types account for >95% of all cases of *K*. *kingae* invasive disease, suggesting that these two polysaccharide structures may have unique properties related to virulence. Given the widespread success of polysaccharide capsule-based vaccines in preventing invasive bacterial disease, this study lays the foundation for a promising strategy to prevent *K*. *kingae* disease.

## Introduction

*Kingella kingae* is being recognized increasingly as an important cause of bone and joint infections in young children, reflecting more sensitive cultivation techniques and increased availability of molecular-based diagnostic tools [1, 2]. Among the key surface factors expressed by *K*. *kingae* is a polysaccharide capsule [3, 4]. Capsules are recognized as important virulence factors in many gram-positive and gram-negative bacteria and have a variety of functions, including inhibiting complement deposition, reducing phagocytosis, and preventing desiccation [5–7]. Polysaccharide capsules conjugated to an immunogenic carrier protein also serve as effective vaccine antigens and have dramatically reduced morbidity and mortality caused by bacteria such as *Streptococcus pneumoniae* [8], *Haemophilus influenzae* type b [9], and *Neisseria meningitidis* [10].

In previous work, we described the structure of the capsule expressed by *K*. *kingae* strain 296–492 as a polymer of [3)-β-Gal*p*NAc-(1→5)-β-Kdo*p*-(2→] and identified the genes essential for capsule synthesis, assembly, and export [3, 11]. In the course of this work, we established that the CsaA glycosyltransferase contains both a GalNAc-transferase domain and a Kdo-transferase domain and is sufficient for creating both the β-Gal*p*NAc-(1→5)-β-Kdo*p* linkage and the β-Kdo*p*-(2→3)-β-Gal*p*NAc linkage. In addition, the CsaA glycosyltransferase may catalyze addition of β-Gal*p*NAc to the terminal β-Kdo residue of the poly-β-Kdo linker [3]. Bendaoud et al. recently reported that the structure of the capsule isolated from the surface of another *K*. *kingae* strain is a polymer of [6)-α-Glc*p*NAc-(1→5)-β-Kdo*p*-(2→] [12], suggesting the existence of at least two different *K*. *kingae* capsule types. The presence of multiple capsule types is well documented in a variety of bacterial species, with examples including *S*. *pneumoniae*, *N*. *meningitidis*, *H*. *influenzae*, and *Klebsiella pneumoniae*. In some cases, specific capsule types are associated more commonly with carriage or more commonly with invasive disease. For example, there are at least 90 different *S*. *pneumoniae* capsule types, but 23 types account for more than 90% of invasive pneumococcal disease worldwide [13, 14]. Similarly, in *N*. *meningitidis* 6 of the 13 characterized capsule types are responsible for 90% of invasive disease cases globally [15, 16]. In *K*. *pneumoniae*, capsule types 1, 3, and 4 are associated with respiratory tract infection, and capsule types 9 and 10 are associated with urinary tract infection [17].

In this study, we set out to define the genetic and structural basis of capsule diversity in a large collection of *K*. *kingae* clinical isolates from Israel. In addition, we examined the relationship between specific capsule types and clinical presentations.

## Results

### Four capsule synthesis loci are present in a diverse collection of *K*. *kingae* clinical isolates

**Fig 1:**
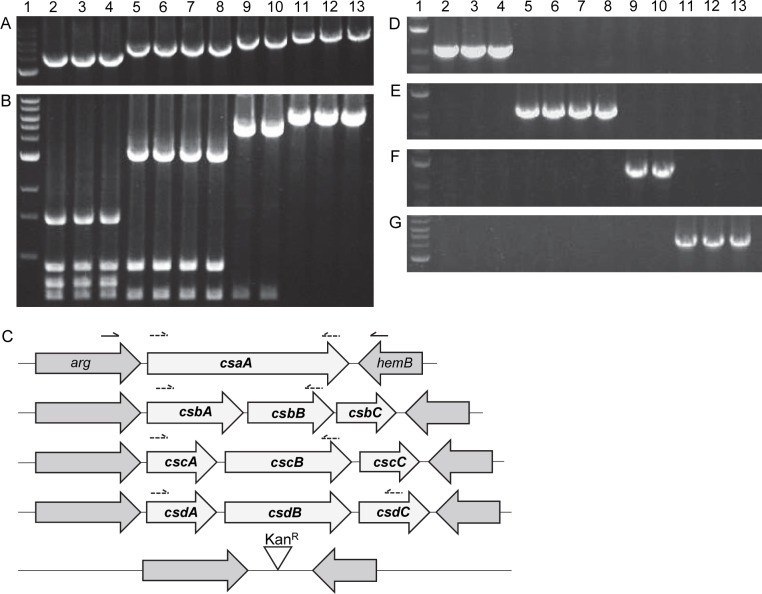
PCR screening of capsule synthesis genes reveals four loci. (A) PCR amplification across the variable synthesis region using flanking primers produces four amplicon sizes. (B) NruI digest of *arg/hemB* amplicon. (C) Illustration of the four capsule synthesis loci and the engineered empty locus. Each locus shows the capsule synthesis genes unique to each capsule type (white) and the highly homologous flanking regions shared among all strains (gray). Solid arrows (size not to scale) denote the approximate location of the screening primers that anneal to the homologous flanking regions used to amplify across the flanking genes irrespective of internal sequence (as shown in Panel A). The dashed arrows above each locus denote the approximate location of the locus-specific screening primers that generate amplicons for (D) the *csa* locus (~2400 bp), (E) the *csb* locus (~2200 bp), (F) the *csc* locus (~2750 bp), and (G) the *csd* locus (~4100 bp). Lane 1, ladder; lane 2, KK01; lane 3, PYKK98; lane 4, PYKK93; lane 5, PYKK89; lane 6, PYKK121; lane 7, PYKK58; lane 8, PYKK59; lane 9, PYKK60; lane 10, D7674; lane 11, E3339; lane 12, D7453; lane 13, BB270.

In initial experiments, we screened a collection of 417 Israeli invasive and carrier isolates for the presence of *csaA*, the capsule synthesis gene in our prototype *K*. *kingae* strain KK01. Using *csaA*-specific primers and PCR, we found that only 47 percent of all isolates contained the *csaA* gene. We hypothesized that other capsule types exist and that the region containing *csaA* represents the *K*. *kingae* capsule synthesis locus and differs in genetic content depending on the enzymatic machinery required to synthesize a specific capsule polysaccharide structure. To test this hypothesis, we designed a forward primer annealing to *arg*, the gene upstream of *csaA* in strain KK01, and a reverse primer annealing to *hemB*, the gene downstream of *csaA* in strain KK01, to amplify across the suspected capsule synthesis locus. As shown in Fig 1A, amplification across this locus in a group of representative isolates in the collection yielded four different amplicon sizes. Restriction mapping of these amplicons with NruI revealed similar banding patterns for strains with the same amplicon size (Fig 1B). Nucleotide sequencing of the amplicons from multiple strains with the same amplicon size revealed an absolute correlation between the amplicon size and the gene content, indicating that the four discrete amplicons represent four discrete loci (Fig 1C). After determining the predicted open reading frames (ORFs) in each amplicon, we searched for the presence of predicted domains or motifs using BLASTP and PHYRE2. The ~3500 bp amplicon contained only the *csaA* gene (identical to our prototype strain KK01) and was named the *csa* locus. The ~4000 bp amplicon contained a gene encoding a predicted GT-B type glycosyltransferase with homology to a GlcNAc transferase (designated *csbA*), a gene encoding a putative capsule synthesis enzyme with homology to a Kdo transferase (designated *csbB*), and a gene encoding a putative enzyme with homology to an acetyltransferase (designated *csbC*) and was named the *csb* locus. The ~5000 bp amplicon contained two genes encoding putative enzymes with homology to halo-acid dehydrogenases (designated *cscA* and *cscB*) and a gene encoding a predicted glycosyltransferase (designated *cscC*) and was named the *csc* locus. The ~5500 bp amplicon contained a gene encoding a predicted galactosyltransferase (*csdA*), a gene encoding a predicted GlcNAc transferase (*csdB*), and a gene encoding a predicted GT-A type glycosyltransferase (*csdC*) and was named the *csd* locus. Based on the sequence of the four unique loci, specific internal primer pairs were generated, producing locus-specific amplicons, as shown in Fig 1D–1G.

### The four capsule synthesis loci are associated with four different polysaccharide capsule types

To confirm that each of the four capsule synthesis loci is associated with a specific capsule type, we examined the glycosyl composition of purified polysaccharide capsule from representative strains that contain either the *csa*, *csb*, *csc*, or *csd* locus. In order to eliminate contamination with the galactan exopolysaccharide produced by *K*. *kingae*, we first deleted the *pam* locus from these strains [11, 18]. As summarized in Table 1, strains KK01, PYKK98, and PYKK93 harbor the *csa* locus and produce a capsule containing GalNAc and Kdo, which we named capsule type a. Strains PYKK89, PYKK121, PYKK58, and PYKK59 harbor the *csb* locus and produce a capsule that contains GlcNAc and Kdo, which we named capsule type b. Strains PYKK60 and D7674 harbor the *csc* locus and produce a capsule that contains ribose and Kdo, which we named capsule type c. Finally, strains E3339, D7453, and BB270 contain the *csd* locus and produce a capsule that contains galactose and GlcNAc, which we named capsule type d. Considered together, these findings demonstrate complete agreement between the capsule synthesis locus and capsule glycosyl composition, indicating that genetic screening of the capsule synthesis locus is predictive of capsule type.

**Table 1:** Association between capsule locus screening and capsule composition.

Clinical isolate	Clinical syndrome	Clonal group	PCR result	GalNAc	GlcNAc	Ribose	Gal	Kdo
KK01	Septic arthritis	H	*csaA*	•				•
PYKK98	Bacteremia/LTB*	B	*csaA*	•				•
PYKK93	Bacteremia	P	*csaA*	•				•
PYKK89	Bacteremia	K	*csbABC*		•			•
PYKK121	Bacteremia	K	*csbABC*		•			•
PYKK58	Septic arthritis	N	*csbABC*		•			•
PYKK59	Bacteremia	N	*csbABC*		•			•
PYKK60	Endocarditis	D	*cscABC*			•		•
D7674	Carrier	R	*cscABC*			•		•
E3339	Carrier	F	*csdABC*		•		•	
D7453	Carrier	G	*csdABC*		•		•	
BB270	Carrier	U	*csdABC*		•		•	

* LTB is laryngotracheobronchitis.

### Structural analysis reveals distinct polysaccharide capsule structures

In previous work, we reported that the type a polysaccharide capsule is a polymer of [3)-β-Gal*p*NAc-(1→5)-β-Kdo*p*-(2→] [11]. To determine the chemical structure of the *K*. *kingae* type b, type c, and type d capsules, surface polysaccharide was purified from derivatives of strains PYKK58 (type b), PYKK60 (type c), and BB270 (type d) lacking the *pam* locus and was analyzed with a combination of linkage analysis and 1-D and 2-D NMR spectroscopy.

**Fig 2:**
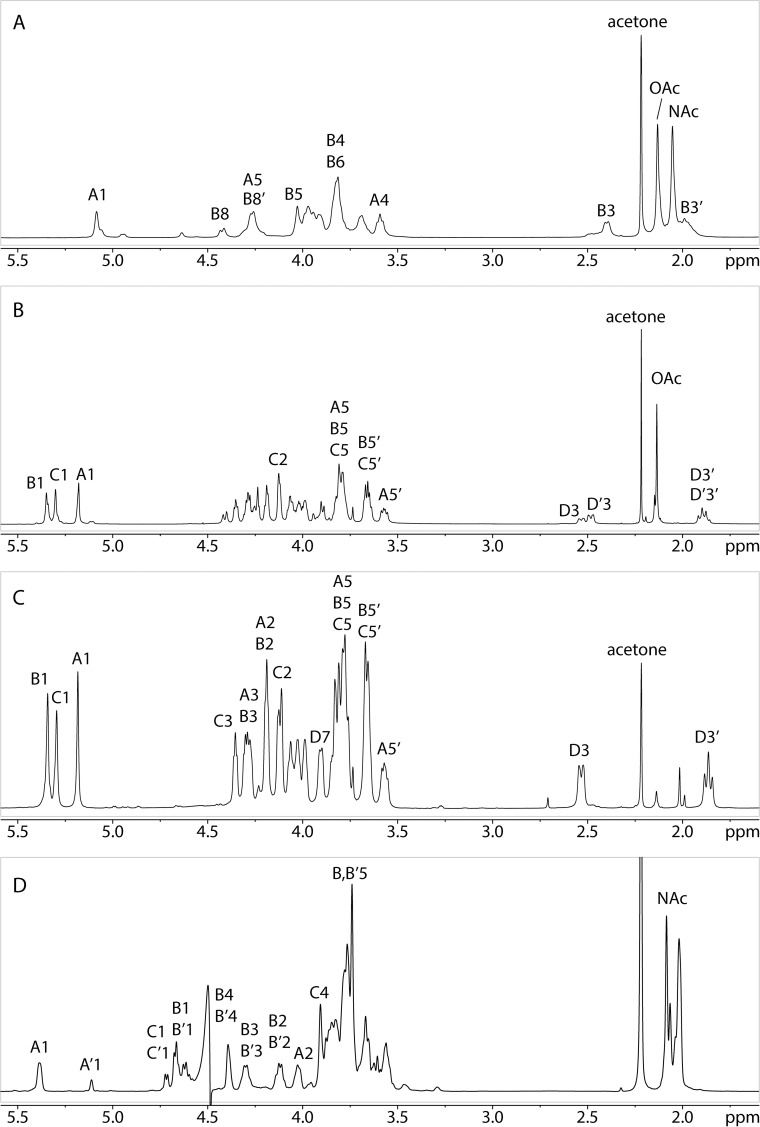
One-dimensional 1H-NMR spectra. The one-dimensional 1H-NMR spectra of the type b (A), type c (B), de-O-acetylated type c (C), and type d (D) polysaccharides are shown.

**Fig 3:**
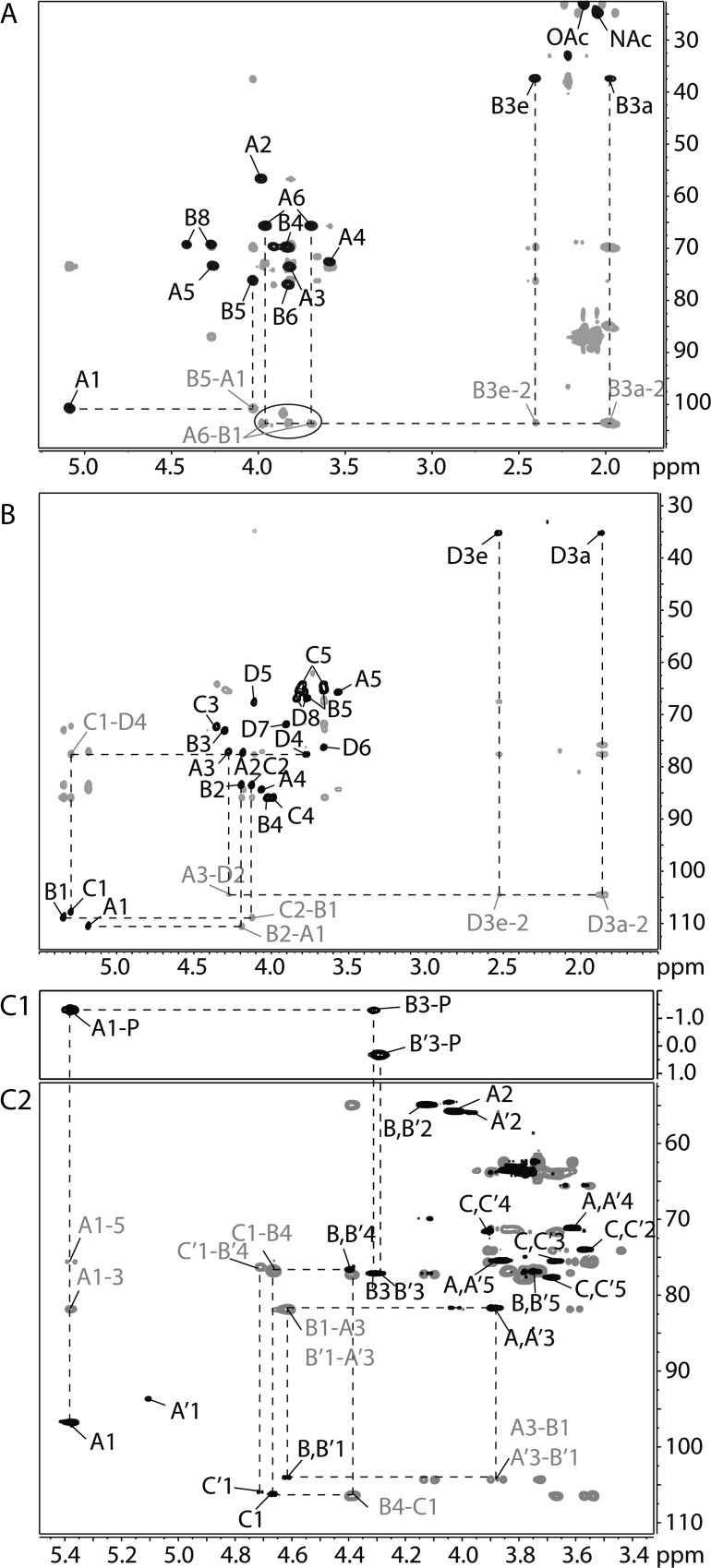
Two-dimensional NMR spectra of the polysaccharides isolated from *K*. *kingae* clinical isolates. (A) Overlay of 2-D ^1^H-^13^C-HMQC (black) and HMBC (gray) NMR spectra of the type b capsule polysaccharide purified from the surface of PYKK58. The circled area is shown at a lower contour level because the peaks in this region were low in intensity. (B) Overlay of 2-D ^1^H-^13^C-HSQC (black) and HMBC (gray) NMR spectra of the type C capsule polysaccharide purified from the surface of PYKK60. Since the Kdo residue does not have an anomeric proton, the HMBC cross peak from H3 to C2 is used to reference the Kdo anomeric carbons in (A) and (B). (C1) ^1^H-^31^P-HMQC spectrum of the type d capsule polysaccharide purified from the surface of BB270. This spectrum shows that the polysacccharide consisting of Residues A, B, and C contains a phosphodiester linking together O-1 of A and O-3 of B and that the sequence consisting of Residues A’, B’, and C’ contains a phosphomonoester attached to O-3 of B’. (C2) Overlay of 2-D ^1^H-^13^C-HSQC (black) and HMBC (gray) NMR spectra of the type d capsule polysaccharide purified from the surface of BB270. Dotted lines and gray labels indicate the inter-residue HMBC correlations showing the connections between residues and thus specifying the sequence of the polysaccharide.

Linkage analysis of the type b capsule gave 1,5,6-tri-O-acetyl-2-deoxy-2-methylacetamido-3,4-di-O-methyl-1-^2^H-glucitol, derived from 6-linked Glc*p*NAc, and 1,2,5,6-tetra-O-acetyl-3-deoxy-4,7,8-tri-O-methyl-1,1,2-tri-^2^H-octitol, derived from 5-linked Kdo (S1A and S2A Figs). Absolute configuration analysis gave D-GlcNAc. Characteristic peaks in the 1-D proton spectrum (Fig 2A) included one major anomeric signal at 5.08 ppm, two signals corresponding to the H-3 protons of Kdo, one N-acetyl peak from GlcNAc, and one O-acetyl of unknown origin. Tracing the connectivities of GlcNAc from H-1 and of Kdo from H-3 in the COSY and TOCSY spectra together with the carbon chemical shifts derived from the HSQC spectrum led to the complete assignment of the chemical shifts belonging to each residue (Table 2 and Fig 3A). Due to the high molecular weight of the sample, the peaks in the spectra were broad and not suitable to measure proton-proton coupling constants for the determination of the anomeric configurations of GlcNAc and Kdo. However, the proton and carbon chemical shifts of the GlcNAc residue agreed with the α-configuration. Comparison of the chemical shifts of the Kdo residue with literature values [19] showed that Kdo was in the β-configuration. The downfield displacement of carbon chemical shifts GlcNAc-C6 and Kdo-C5 indicated the linkage positions as 6-linked GlcNAc and 5-linked Kdo. The downfield displacement of the proton chemical shifts of Kdo-H8 together with the intensity (3H) and chemical shifts of the O-acetyl signal (2.13/23.1 ppm) indicated acetylation on O-8 of Kdo. Taken together, these results indicated that the polymer is composed of a disaccharide repeating unit with the structure [6)-α-D-Glc*p*NAc-(1→5)-β-(8-OAc)Kdo*p*-(2→].

**Table 2:** Chemical shift assignments of the type b capsular polysaccharide purified from PYKK58.

No.	Residue	Chemical shift (ppm)								NOE
		1	2	3	4	5	6	7	8	*HMBC*
A	6-α-Glc*p*NAc	5.08	3.99	3.81	3.59	4.26	3.96/3.70			B5
		***100*.*9***	*56*.*9*	*73*.*6*	*72*.*8*	*73*.*2*	***65*.*9***			*B5*
B	8-OAc-5-β-Kdo	-	-	1.97/2.42	3.83	4.02	3.83	3.91	4.42/4.27	
		*175*.*0*	***103*.*3***	*37*.*5*	*69*.*9*	***76*.*2***	*77*.*1*	*69*.*7*	*69*.*3*	*A6*

Additional signals: OAc: 2.13/23.0 ppm; NAc: 2.05/24.7 ppm

Carbon chemical shifts are in italics, and carbon resonance that are shifted downfield due to glycosylation are in bold.

Linkage analysis of the type c capsule gave 1,3,4-tri-O-acetyl-2,5-di-O-methyl-1-^2^H-ribitol, derived from 3-linked ribofuranose, 1,2,4-tri-O-acetyl-3,5-di-O-methyl-1-^2^H-ribitol, derived from 2-linked ribofuranose, and 1,2,4,6-tetra-O-acetyl-3-deoxy-5,7,8-tri-O-methyl-1,1,2-tri-^2^H-octitol, derived from 4-linked Kdo (S1B and S2B Figs). Absolute configuration analysis gave D-ribose. Characteristic peaks in the 1-D proton NMR spectrum (Fig 2B) included three anomeric signals at 5.34, 5.29, and 5.18 ppm, two pairs of signals corresponding to the H-3 protons of Kdo, an acetyl methyl signal, and several resonances in the carbohydrate ring region. The presence of two sets of Kdo signals of unequal intensity (ratio 2:3) together with the presence of an acetyl signal with an area three times that of the larger Kdo-H3 peak suggested that 60% of the Kdo residues in the polysaccharide were O-acetylated. To reduce the heterogeneity of the sample, we performed de-O-acetylation. The 1-D proton NMR spectrum of the de-O-acetylated material (Fig 2C) was simplified compared to the native polysaccharide and displayed only a single set of Kdo H-3 peaks. Tracing the connectivities of the three anomeric signals from H-1 and of Kdo from H-3 in the COSY and TOCSY spectra together with the carbon chemical shifts derived from the HSQC spectrum led to a complete chemical shift assignment and revealed the presence of two 2-linked and one 3-linked ribofuranose residues as well as one 4-linked Kdo residue (Table 3 and Fig 3B). The proton and carbon chemical shifts of the ribose residues agreed with β-anomeric configuration [20, 21]. Comparison of the chemical shifts of the Kdo residue with literature values [19] showed that Kdo also had the β-configuration. The NOESY (S3 Fig) and HMBC (Fig 3B) spectra showed inter-residue cross peaks, allowing the determination of the sequence of the four monosaccharide residues in the polysaccharide repeating unit. Thus, the three ribose anomeric protons were correlated with their respective non-reducing end neighbors in both NOESY and HMBC spectra, and C-2 of Kdo (and of 8-OAc-Kdo) was correlated in HMBC to H-3 of Residue C. Taken together, these results indicated that the polymer is composed of a tetrasaccharide repeating unit with the structure [3)-β-D-Rib*f*-(1→2)-β-D-Rib*f*-(1→2)-β-D-Rib*f*-(1→4)-β-Kdo*p*-(2→].

**Table 3:** Chemical shift assignments of the type c capsular polysaccharide purified from PYKK60.

No.	Residue	Chemical shift (ppm)								NOE
		1	2	3	4	5	6	7	8	*HMBC*
A	3-β-Rib*f*	5.18	4.18	4.28	4.06	3.79/3.57				B2
		***110*.*4***	*77*.*3*	***77*.*3***	*84*.*4*	*65*.*7*				*B2*
B	2-β-Rib*f*	5.34	4.19	4.30	4.02	3.82/3.66				C2
		***108*.*9***	***83*.*5***	*73*.*1*	*86*.*0*	*65*.*4*				*C2*
C	2-β-Rib*f*	5.29	4.12	4.35	3.98	3.80/3.66				D4,5
		***107*.*8***	***83*.*5***	*72*.*4*	*85*.*9*	*64*.*4*				*D4*
D	4-β-Kdo	-	-	2.53/1.85	3.77	4.11	3.66	3.91	3.83/3.77	
		*175*.*9*	***104*.*5***	*35*.*1*	***77*.*7***	*67*.*7*	*76*.*3*	*71*.*9*	*66*.*9*	*A3*
D’	8-OAc-4-β-Kdo	-	-	2.48/1.89	3.80	4.13	3.89	4.05	4.40/4.27	
		*175*.*5*	***104*.*3***	*35*.*0*	***77*.*4***	*67*.*6*	*76*.*2*	*69*.*9*	*69*.*0*	*A3*

Additional signals: OAc: 2.14/23.2 ppm

Carbon chemical shifts are in italics, and carbon resonance that are shifted downfield due to glycosylation are in bold.

The main PMAA derivatives found in the linkage analysis of type d capsule were 1,5-di-O-acetyl-2,3,4,6-tetra-O-methyl-1-^2^H-galactitol, derived from terminal galactopyranose, and 1,3,5-tri-O-acetyl-2-deoxy-2-methylacetamido-4,6-di-O-methyl-1-^2^H-glucitol, derived from 3-linked Glc*p*NAc (S1C Fig). Absolute configuration analysis gave D-GlcNAc and D-galactose. The 1-D proton spectrum (Fig 2D) included two α-anomeric signals (ratio 3.4:1), a cluster of several β-anomeric signals, and a group of N-acetyl peaks. The β-anomeric cluster was resolved into four distinct resonances in the HSQC spectrum. Tracing the connectivities from these anomeric signals in the COSY and TOCSY spectra together with the carbon chemical shifts obtained from the HSQC spectrum allowed a complete assignment of a total of six different residues that were grouped into three pairs of residue types (Table 4 and Fig 3C2). The chemical shifts of two of the six residues identified them as 3-linked α-GlcNAc (A and A’), another two of the six as 3,4-linked β-GlcNAc (B and B’), and the final two of the six as terminal β-Gal (C and C’). This information suggested the presence of two similar trisaccharide repeating units in the polysaccharide. Inter-residue linkages were assigned from HMBC and NOESY correlations. The HMBC (Fig 3C2) and NOE correlations (S4 Fig) between H1 of B/B’ and H3 of A/A’ and between H1 of C/C’ and H4 of B/B’ confirmed the presence of two slightly different 3)-[β-Gal-(1→4)]-β-GlcNAc-(1→3)-α-GlcNAc-(1→ trisaccharides, but there were no HMBC or NOE correlations to H1 of A or H3 of B that would link these trisaccharides together. The unusual downfield shifts of A-H1, B-H3, and B’-H3 suggested substitution by an electronegative group, such as acetate, sulfate, or phosphate. The fact that no significant amount of 3,4-linked GlcNAc was detected in the linkage analysis supports the presence of phosphate, as a phosphorylated PMAA would not be detected in GC-MS due to low volatility. Indeed, ^31^P NMR showed signals at 0.42 and -1.20 ppm, consistent with phosphomono- and diesters, respectively (S5 Fig). A 2D-^1^H-^31^P-HMQC spectrum confirmed the presence of phosphodiester and its attachment to O1 of Residue A and O3 of Residue B and of a phosphomonoester and its attachment to O3 of Residue B’ (Fig 3C1). Taken together, these results strongly indicated that the polymer is composed of a trisaccharide repeating unit with the structure [P-(O→3)[β-D-Gal*p*-(1→4)]-β-D-Glc*p*NAc-(1→3)-α-D-Glc*p*NAc-1-], whereby Residues A, B, and C make up repeating units at the non-reducing end and the interior of the polysaccharide and Residues A’, B’, and C’ constitute the reducing end trisaccharide repeat. The intensity ratio (3.4:1) of the anomeric signals of A and A’ points to an average of about 4–5 repeating units per polysaccharide chain. This conclusion was confirmed by SEC and NSI-MS, which showed the presence of polysaccharide chains consisting of a small number of repeating units (S6 Fig).

**Table 4:** Chemical shift assignments of the type d capsular polysaccharide purified from BB270.

No.	Residue	Chemical shift (ppm)						NOE
		1	2	3	4	5	6	*HMBC*
A	3-α-Glc*p*NAc	5.39	4.03	3.88	3.61	3.85	3.81/3.75	
		*96*.*7*	*55*.*6*	***81*.*6***	*71*.*1*	*75*.*4*	*63*.*5*	
A'	3-α-Glc*p*NAc	5.11	3.96	3.87	3.56	3.85	3.82/3.76	
		*93*.*8*	*55*.*8*	***81*.*6***	*71*.*5*	*75*.*5*	*63*.*5*	
B	3,4-β-Glc*p*NAc	4.62	4.13	4.30	4.39	3.75	3.84/3.77	A3
		***104*.*1***	*54*.*6*	***77*.*0***	***76*.*6***	*76*.*9*	*63*.*4*	*A3*
B'	3,4-β-Glc*p*NAc	4.60	4.11	4.28	4.38	3.75	3.84/3.77	A'3
		***104*.*1***	*54*.*6*	***77*.*0***	***76*.*6***	*76*.*9*	*63*.*4*	*A’3*
C	t-β-Gal*p*	4.68	3.57	3.66	3.91	3.68	3.77/3.75	B4
		***106*.*3***	*71*	*75*.*4*	*71*.*4*	*77*.*8*	*63*.*8*	*B4*
C'	t-β-Gal*p*	4.72	3.58	3.67	3.91	3.68	3.77/3.75	B'4
		***106*.*1***	*73*.*9*	*75*.*6*	*71*.*4*	*77*.*8*	*63*.*8*	*B’4*

Additional signals: NAc: 2.09/25.1 ppm; 2.07/25.0 ppm; 2.03/25.6 ppm; 2.10/25.4 ppm

Carbon chemical shifts are in italics, and carbon resonance that are shifted downfield due to glycosylation are in bold.

**Fig 4:**
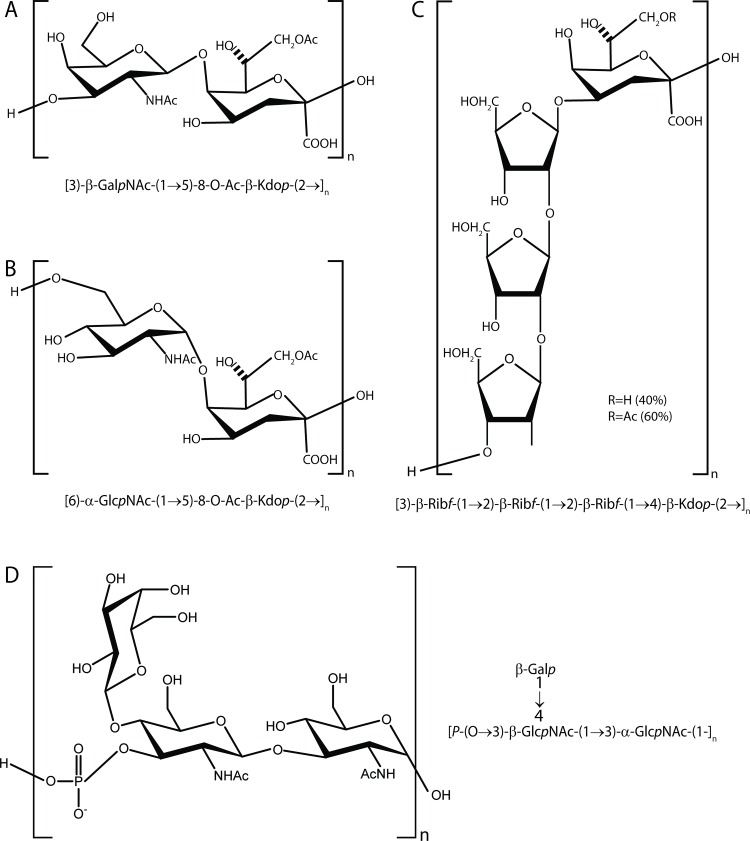
Capsule polysaccharide repeating unit structures. The capsule polysaccharide repeating unit structures for capsule type a (GalNAc-Kdo, panel A), capsule type b (GlcNAc-Kdo, panel B), capsule type c (ribose-Kdo, panel C), and capsule type d (galactose-GlcNAc, panel D) are shown.

NMR spectra and chemical shift assignments for the type b, c, and d capsules are shown in Fig 3A–3C and Tables 2–4. Structural analysis revealed that the type b capsule structure is a polymer of [6)-α-D-Glc*p*NAc-(1→5)-β-(8-OAc)Kdo*p*-(2→], the type c capsule structure is a polymer of [3)-β-D-Rib*f*-(1→2)-β-D-Rib*f*-(1→2)-β-D-Rib*f*-(1→4)-β-Kdo*p*-(2→], and the type d capsule structure is a polymer of [P-(O→3)[β-D-Gal*p*-(1→4)]-β-D-Glc*p*NAc-(1→3)-α-D-Glc*p*NAc-1-]. A comparison of the structures of the type a, type b, type c, and type d capsules is shown in Fig 4.

### The *csa*, *csb*, *csc*, and *csd* capsule synthesis loci are necessary and sufficient for polysaccharide capsule synthesis

**Fig 5:**
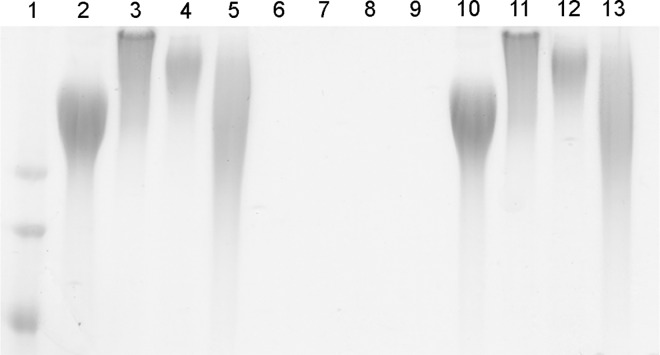
Comparison of capsule migration pattern between capsule types. Alcian blue stained gel depicting the migration pattern of capsule material purified from the surface of the source strains (lanes 2–5), capsule locus deletion mutants (lane 6–9), and the capsule complements (lanes 10–13). Lane 1, ladder; lane 2, KK01; lane 3, PYKK58; lane 4, PYKK60; lane 5, BB270; lane 6, KK01Δ*csa*; lane 7, PYKK58Δ*csb*; lane 8, PYKK60Δ*csc*; lane 9 BB270Δ*csd*; lane 10, KK01Δ*csa(csa)*; lane 11 PYKK58Δ*csb(csb)*; lane 12, PYKK60Δ*csc(csc)*; lane 13 BB270Δ*csd(csd)*.

To confirm that the *csa*, *csb*, *csc*, and *csd* loci are essential for production of capsule, we deleted each of these loci and then examined the resulting strains for surface material that stains with Alcian blue. As shown in Fig 5, targeted deletion of the *csa*, *csb*, *csc*, or *csd* locus resulted in loss of surface extractable capsule from strains KK01, PYKK58, PYKK60, and BB270, respectively. Chromosomal complementation of each of these regions at the native locus resulted in restoration of encapsulation. These results demonstrate that the capsule synthesis loci are essential for production of capsule in representative type a, type b, type c, and type d *K*. *kingae* strains.

**Fig 6:**
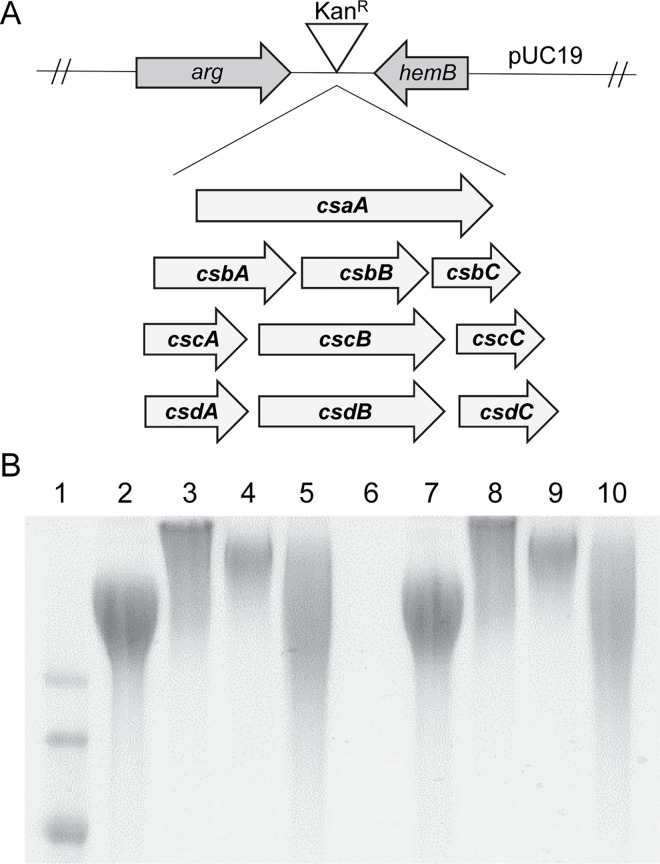
**(A) Illustration of the capsule swap vector in pUC19 harboring the *csa*, *csb*, *csc*, or *csd* locus with a Kan** ^**R**^ **marker for selection, and (B) the migration patterns of capsule material from isogenic capsule swaps.** Alcian blue stained gel depicting the migration pattern of capsule material purified from the surface of the source strains (lanes 2–5) and the capsule swaps expressed in the isogenic KK01 background (lanes 7–10). Lane 1, ladder; lane 2, KK01; lane 3, PYKK58; lane 4, PYKK60; lane 5, PYKKBB270; lane 6, KK01Δ*csa*; lane 7, KK01Swap*csa*; lane 8, KK01Swap*csb*; lane 9, KK01Swap*csc*; lane 10, KK01Swap*csd*.

In additional experiments, we examined the ability of the type a, b, c, and d loci to complement a deletion of the *csa* locus in prototype strain KK01 and produce the corresponding capsule. In performing these studies, we engineered a deletion of *csaA* with no effect on the flanking *arg* and *hemB* genes, producing a strain called KK01Δ*csa*. Subsequently, we generated a plasmid called pSwap, which contains the *arg* and *hemB* genes, a kanamycin resistance marker, and a partial pUC19 multiple cloning site (MCS) (Fig 6A). Using this plasmid, we inserted each of the four capsule synthesis loci into the MCS, generating pSwap*csa*, pSwap*csb*, pSwap*csc*, and pSwap*csd*. Each of these plasmids was linearized and transformed into strain KK01Δ*csa*, producing strains KK01Swap*csa*, KK01Swap*csb*, KK01Swap*csc*, and KK01Swap*csd*. As shown in Fig 6B, each of these strains produced a capsule as assessed by Alcian blue staining of surface extracts. To confirm that the capsule in each of these strains corresponded to the specific capsule synthesis locus, surface polysaccharide was extracted and examined initially by Alcian blue staining. As expected, the Alcian blue staining profile of the capsule extracted from the *csa*, *csb*, *csc*, or *csd* swap strains was similar to the profile of the parental capsule locus source strain, suggesting that the capsules produced in a KK01Δ*csa* background strain retain their native migration pattern (Fig 6B). 1-D Proton NMR analysis demonstrated that strain KK01Swap*csa* produced the type a capsule, strain KK01Swap*csb* produced the type b capsule, strain KK01Swap*csc* produced the type c capsule, and strain KK01Swap*csd* produced the type d capsule (Table 5). These results demonstrate that the *csa*, *csb*, *csc*, and *csd* loci encode the synthesis components of the four *K*. *kingae* capsule types and are functional in an isogenic strain background containing the capsule export and assembly machinery [3, 4].

**Table 5:** Comparative molar ratio of main glycosyl residues in polysaccharide purified from the surface of the capsule swap strains as detected by 1-D Proton NMR.

Sample	β-Ribf	α-GlcNAc	β-GalNAc	β-Gal	β-Kdo
KK01Swap*csa*	---	---	1.23	---	1.0
KK01Swap*csb*	---	1.36	---	---	1.0
KK01Swap*csc*	4.65	---	---	---	1.0
KK01Swap*csd*	---	1.7	---	1.0	---

### The type a and type b capsules are enriched in invasive isolates of *K*. *kingae*

With our knowledge of the type a, type b, type c, and type d capsule loci in hand, we used a PCR approach to examine a large collection of *K*. *kingae* clinical isolates for capsule type. A total of 417 Israeli strains isolated between 1990 and 2014 were investigated. The collection contains 239 strains isolated from healthy pharyngeal carriers and 178 strains recovered from patients with a variety of invasive infections, including skeletal system infections, bacteremia, and endocarditis, allowing characterization of the capsule types elaborated by the full range of *K*. *kingae* isolates in the country. Overall, 413 of the 417 (99.0%) strains were genotyped by pulsed-field gel electrophoresis (PFGE) [22] and found to belong to 60 distinct clones, including 16 clones that were represented in the collection by ≥7 strains and that collectively accounted for 345 (83.5%) of all typed strains.

One of the four capsule synthesis loci was identified in all of the strains, except for strain KK183 belonging to PFGE clonal group Tnc, which was isolated from the synovial fluid of a child with septic arthritis. This strain was shown to be nonencapsulated and did not generate capsule locus flanking or capsule locus specific PCR amplicons (S7 Fig). Therefore, this strain was not included in the analysis of the association between capsule type and invasiveness or clonal distribution. A second nonencapsulated strain (KK56, PFGE clone S) was isolated from a child with arthritis and was found to have a *csaA* gene with a 512 bp internal deletion in the ORF, which is predicted to introduce a frameshift mutation leading to a truncated CsaA protein (S7 Fig). This strain was included among organisms with capsule type a for the purposes of the data analysis.

**Fig 7:**
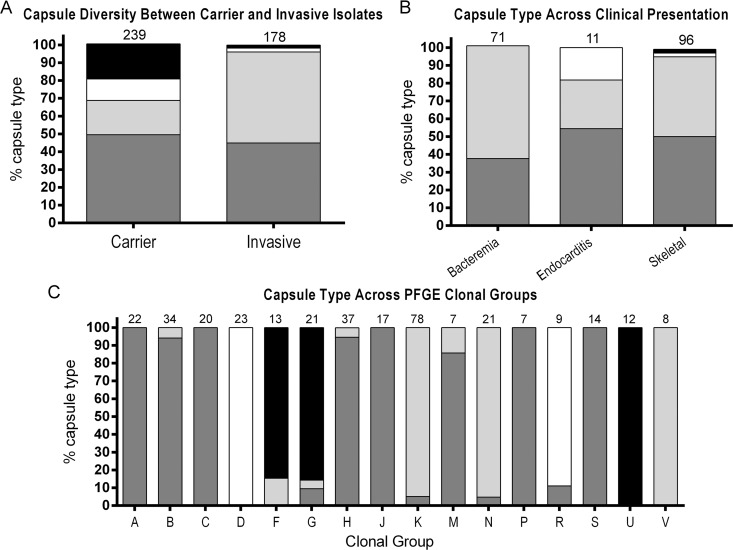
Capsule type diversity in *K*. *kingae* clinical isolates. Type a is shown in dark gray, type b in light gray, type c in white, and type d in black. The number above each bar represents the number of isolates in each group. (A) The capsule type representation among carrier isolates (type a, 49.0%; type b, 19.2%; type c, 12.1%; type d, 19.7%) and invasive isolates (type a, 44.9%; type b, 51.5%; type c, 2.2%; type d, 1.7%) is shown. (B) The capsule type representation among common *K*. *kingae* clinical presentations is shown: bacteremia (type a, 37.7%; type b, 63.3%; type c, 0%; type d, 0%), endocarditis (type a, 54.5%; type b, 27.3%; type c, 18.2%; type d, 0%), and skeletal infections (type a, 50.0%; type b, 44.8%; type c, 2.1%; type d, 1.1%). (C) The capsule types among PFGE clonal groups containing ≥7 isolates are shown. Capsule type was determined by PCR screening for each of the four capsule synthesis loci.

While capsule type a was common among both carrier and invasive isolates, the distribution of capsule types b, c, and d in the invasive versus carrier groups showed statistically significant differences using the χ^2^ test (*P<0*.*001*; Fig 7A and S1 Table). Overall, capsule type a or type b was found in 171 of 178 (96.1%) invasive strains but in only 163 of 239 (68.2%) carrier strains (*P<0*.*001*). Employing capsule type d as the reference, the logistic regression showed that capsule type a had an OR of 15.9 for invasive disease (*P<0*.*001*, 95% CI: 3.8–67.5), capsule type b had an OR of 48.0 for invasive disease (*P<0*.*001*, 95% CI: 11.2–206.7), and capsule type c had an OR of 3.2 for invasive disease (*P = 0*.*346*, 95% CI: 0.4–15.4).

The different capsule types showed significant associations with specific invasive clinical syndromes using the χ^2^ test (*P<0*.*001*). While capsule type b was more frequent among cases of bacteremia (45 of 71, 63.4%), capsule type a was found in one-half (48 of 96) of skeletal system infections (Fig 7B and S2 Table).

Statistical analysis using the χ^2^ test demonstrated a significant association of capsule type and PFGE clones (*P<0*.*001*). Of note, all 16 common PFGE clones showed a clear predominance of a single capsule type: capsule type a in PFGE clones A, B, C, H, J, M, P, and S; capsule type b in PFGE clones K, N, and V; capsule type c in PFGE clones D and R; and capsule type d in PFGE clones F, G, and U (Fig 7C and S3 Table).

## Discussion

In this study we examined a large collection of *K*. *kingae* clinical isolates and established that there are four different *K*. *kingae* capsule types. In addition, we identified the underlying capsule synthesis genes for each capsule type. Using a combination of mass spectroscopy and NMR, we also determined the structure of two previously uncharacterized capsule types (type c and type d), complementing previous work on the structure of the type a capsule and the type b capsule [11, 18]. Finally, we used a genetic screen to determine the capsule type of invasive disease isolates and healthy carrier isolates and discovered that capsule type a and type b account for 96% of all invasive disease isolates and that capsule type c and type d are disproportionately present among healthy carrier isolates.

At the outset of our study, we hypothesized that our large collection of *K*. *kingae* clinical isolates would contain multiple polysaccharide capsule types. Bacterial polysaccharide capsules are traditionally typed using one of two methods: 1) genetically, based on the presence of specific capsule synthesis genes in the capsule locus, or 2) immunologically, based on agglutination reactions using capsule-specific sera. In this study we used a PCR-based genetic screening method, similar to methods that assess the capsule polysaccharide synthesis region for capsule typing of *K*. *pneumoniae* [23–25], *Pasteurella multocida* [26], and *N*. *meningitidis* [27]. Using this approach, we established that there are four different capsule types in *K*. *kingae*, with each strain containing only one of four distinct capsule synthesis loci. 1-D Proton NMR analysis of purified capsule from isogenic capsule synthesis locus swap strains confirmed that capsule type is determined by the gene content of the capsule synthesis locus (Table 5).

The presence of multiple capsule types in a species is well documented for a variety of encapsulated pathogens, with examples including *S*. *pneumoniae* (>90 types), *E*. *coli* (>80 types), *Klebsiella pneumoniae* (78 types), *N*. *meningitidis* (13 types), and *H*. *influenzae* (6 types). Of the four capsule structures that we describe, two have been previously described in other species. In particular, the type a capsule containing [3)-β-Gal*p*NAc-(1→5)-β-Kdo*p*-(2→] is identical to the capsule of *Moraxella nonliquefaciens* strain 3828/60 [28], and the type b capsule containing [6)-α-Glc*p*NAc-(1→5)-β-(8-OAc)Kdo*p*-(2→] is identical to the *Actinobacillus pleuropneumoniae* serotype 5a capsule [18, 29]. In contrast, the type c capsule containing [3)-β-Rib*f*-(1→2)-β-Rib*f*-(1→2)-β-Rib*f*-(1→4)-β-Kdo*p*-(2→] and the type d capsule containing [P-(O→3)[β-Gal*p*-(1→4)]-β-Glc*p*NAc-(1→3)-α-Glc*p*NAc-1-] are novel.

Uropathogenic *E*. *coli* are typically encapsulated with acidic polysaccharides, often containing Kdo together with one or two ribose moieties (di- or tri-saccharide) in the repeating unit. For example, the *E*. *coli* K16-antigen [2)-β-D-Rib*f*-(1→3)-β-D-Rib*f*-(1→5)-α-Kdo*p*-(2→] [30] and the *E*. *coli* K74 antigen [3)-β-D-Rib*f*-(1→2)-β-D-Rib*f*- (l→6)-β-Kdo-(2→] [31] both contain Kdo and ribose in unequal ratios, similar to the type c capsule in *K*. *kingae*. The Kdo-ribose polysaccharides form a group of closely related but serologically distinct *E*. *coli* capsule antigens, and the serologic variability is increased by different degrees of O-acetylation at various sites [32]. We also found acetylation in the *K*. *kingae* type c capsule, with 60% of the R groups being acetylated. The functional consequence of type c capsule acetylation in terms of serological reactivity remains to be investigated.

It is interesting to speculate regarding the potential for interstrain capsule type switching in *K*. *kingae*. In *N*. *meningitidis*, capsule switching has been shown to result from recombination of the polysialyltransferase gene (*siaD*) or the capsule biosynthesis operon [33], with evidence for capsule switching between strains implicated in carriage and strains associated with invasive disease [34]. Pneumococcal isolates can also undergo capsule switching, with the serotype of a clone changing due to alteration in the capsule biosynthesis locus via mutations or through genetic recombination [35–37]. In support of the possibility of intraspecies capsule switching in *K*. *kingae*, several *K*. *kingae* clonal groups are associated with multiple capsule types.

Asymptomatic carriage provides an ideal environment for interspecies exchange of genetic material among bacteria that occupy the same niche [38]. The human nasopharynx has been shown to harbor diverse bacteria, including *N*. *meningitidis*, *H*. *influenzae*, and *S*. *pneumoniae* as well as nonpathogenic *Neisseria* spp. and *Moraxella* spp. Evidence supporting horizontal gene transfer between phylogenetically distant species is seen in the meningococcal genome, which harbors three independent domains of *Haemophilus*-like DNA. Uptake and integration of DNA in the upper respiratory tract is a probable mechanism to explain the capsule diversity observed in *K*. *kingae* in this study. *Actinobacillus* spp., *Moraxella* spp., and *Kingella* spp. are all found in normal human flora of the upper respiratory tract, providing the opportunity for horizontal gene transfer from other genera as the genesis of the four *K*. *kingae* capsule synthesis loci. *M*. *nonliquefaciens* has been shown to be present in the respiratory tract of young children [39, 40]. *A*. *pleuropneumoniae* is primarily a swine pathogen, but other *Actinobacillus* spp. can be found in humans [41].

All of the *K*. *kingae* strains in our collection gave a PCR product for the capsule export and assembly genes *ctrABCD*, *lipA*, and *lipB*, suggesting that all of these strains contain the machinery necessary to display a capsule polymer on their surface [3]. However, out of 417 isolates, two strains demonstrated atypical PCR capsule typing results. First, strain PYKK56 yielded a *csa* locus PCR product, but the product was smaller than expected (S7C Fig). Sequencing of the *csa* locus in this strain revealed a 512 bp internal deletion in the *csaA* gene, resulting in frameshift that is predicted to lead to a truncated CsaA protein (S7G Fig). Alcian blue staining of surface extracts from this strain revealed no capsule, suggesting that the *csaA* mutation resulted in abrogation of capsule expression (S7A Fig). Second, strain PYKK183 yielded no capsule locus flanking product and no capsule locus-specific PCR product (S7B–S7G Fig). Alcian blue staining of surface extracted material revealed that this strain is not encapsulated, suggesting that this strain lacks capsule synthesis genes, rather than possessing a unique capsule synthesis locus (S7A Fig).

Amit et al. determined that *K*. *kingae* PFGE clonal groups B, H, K, N, and P account for 72.9% of all invasive isolates and that PFGE clonal groups A, C, D, F, G, J, R, S, and U are rare among invasive disease isolates [42]. Interestingly, only capsule types a and b are represented in the B, H, K, N, and P clonal group isolates. Overall, the type a and type b capsules account for 96% of invasive isolates but less than 70% of the carrier isolates. The type c capsule is most prevalent in the D and R clonal groups, and the type d capsule is most prevalent in the F, U, and G clonal groups, all of which fall into the subset of rare-disease PFGE clonal groups. The significant difference in the capsule type distribution between strains carried by asymptomatic children and those isolated from patients with invasive infections may suggest that the type c and type d capsules provide incomplete protection to *K*. *kingae* organisms, enabling them to colonize the oropharyngeal epithelium but not allowing their survival in the bloodstream, the skeletal system, or the endocardium. This phenomenon of specific capsule type association with invasive disease is reminiscent of encapsulated *H*. *influenzae*, an upper respiratory tract colonizer that elaborates six distinct polysaccharide capsules, with strains elaborating the type b capsule accounting for almost all cases of disease prior to the introduction of the conjugate vaccines [43]. However, the association between capsule type and virulence may not be causal. Close examination of the data in Fig 7C and S3 Table shows that 49 of 51 (96.1%) strains belonging to the A, C, and M clonal groups, which appear to have diminished virulence and were collectively associated with only 4 of 181 (2.2%) invasive infections in Israel according to a study published in 2012 [42], elaborate polysaccharide capsule types a or b, indicating that determinants other than capsule type likely play an important role in the potential of the organism to cause invasive disease. This possibility is indirectly supported by the fact that two nonencapsulated strains, KK183 and KK56, were able to cause septic arthritis in otherwise healthy children.

Considering the effectiveness of many polysaccharide-conjugate vaccines in reducing childhood morbidity and mortality, it is interesting to speculate that a *K*. *kingae* capsule polysaccharide-conjugate vaccine may be an effective strategy to prevent *K*. *kingae* disease, pending additional analysis of the global burden of *K*. *kingae* disease. While more studies are needed, the discovery that the capsule repertoire of a diverse collection of *K*. *kingae* carrier and invasive disease isolates is represented by only 4 capsule types, with two capsule types accounting for >95% of invasive disease, is an important first step in establishing the feasibility of a vaccine for the prevention of *K*. *kingae* disease.

## Methods

### Bacterial strains and growth conditions

The strains representative of each capsule type that were used for the fundamental studies in the work are listed in Table 6. The complete list of clinical isolates that were examined for capsule type are shown in S4 Table. *K*. *kingae* strain 269–492 was isolated from the joint fluid of a child with septic arthritis at St. Louis Children’s Hospital, St. Louis, MO. *K*. *kingae* strain KK01 is a stable natural variant of strain 269–492 that grows as a non-spreading, non-corroding colony type and was used as the primary strain in this study [44]. *K*. *kingae* and *E*. *coli* strains were grown and stored as previously described [4, 11].

**Table 6:** Strains and plasmids used in this study.

Strain	Description	Source
269–492	*K*. *kingae* isolate from St. Louis Children’s Hospital	[45]
KK01	Nonspreading/noncorroding derivative of 269–492	[45]
PYKK121	Clonal group K isolate from a case of bacteremia	P. Yagupsky
PYKK89	Clonal group K isolate from a case of bacteremia	P. Yagupsky
PYKK93	Clonal group P isolate from a case of bacteremia	P. Yagupsky
PYKK98	Clonal group B isolate from a case of bacteremia	P. Yagupsky
PYKK60	Clonal group D isolate from a case of endocarditis	P. Yagupsky
PYKK58	Clonal group N isolate from a case of septic arthritis	P. Yagupsky
PYKK59	Clonal group N isolate from a case of bacteremia	P. Yagupsky
D7674	Clonal group R isolate from a healthy carrier	P. Yagupsky
E3339	Clonal group F isolate from a healthy carrier	P. Yagupsky
D7453	Clonal group G isolate from a healthy carrier	P. Yagupsky
BB270	Clonal group U isolate from a healthy carrier	P. Yagupsky
KK01Δ*csa*	Contains *csaA* deletion	[3]
PYKK58Δ*csb*	Contains capsule synthesis locus *csbABC* deletion	This study
PYKK60Δ*csc*	Contains capsule synthesis locus *cscABC* deletion	This study
BB270Δ*csd*	Contains capsule synthesis locus *csdABC* deletion	This study
KK01Δ*csa(csa)*	Complement of *csa* locus in KK01Δ*csa*	[3]
PYKK58Δ*csb(csb)*	Complement of *csb* locus in PYKK58Δ*csb*	This study
PYKK60Δ*csc(csc)*	Complement of *csc* locus in PYKK60Δ*csc*	This study
BB270Δ*csd(csd)*	Complement of *csd* locus in BB270Δ*csd*	This study
KK01SwapEmpty	Contains the capsule synthesis locus flanking genes and a deletion of the *csaA* region.	This study
KK01Swap*csa*	KK01SwapEmpty containing the *csa* capsule synthesis locus	This study
KK01Swap*csb*	KK01SwapEmpty containing the *csb* capsule synthesis locus	This study
KK01Swap*csc*	KK01SwapEmpty containing the *csc* capsule synthesis locus	This study
KK01Swap*csd*	KK01SwapEmpty containing the *csd* capsule synthesis locus	This study
***E*. *coli* plasmids**		
pUC19*pam*::*ermC*	*pam* locus deletion plasmid	[11]
pSwapEmpty	Contains the capsule synthesis locus flanking genes with the capsule synthesis variable region deleted	This study
pSwap*csa*	pSwapEmpty with *csa* locus inserted	This study
pSwap*csb*	pSwapEmpty with *csb* locus inserted	This study
pSwap*csc*	pSwapEmpty with *csc* locus inserted	This study
pSwap*csd*	pSwapEmpty with *csd* locus inserted	This study

### Clinical isolate strain collection

*K*. *kingae* isolates were selected from a large assortment of Israeli strains that have been gathered at the Soroka University Medical Center since the early 1990’s. The collection contains isolates from patients with a variety of invasive infections and from healthy pharyngeal carriers in the course of epidemiological studies on *K*. *kingae* carriage and transmission.

A total of >200 *K*. *kingae* invasive strains and >600 *K*. *kingae* carrier isolates have been typed by pulsed field gel electrophoresis (PFGE) [42], and a sample of the predominant PFGE clones has been further characterized by MLST and *rtx*A gene sequencing [46]. Based on genotyping results, strains were selected to meet the following study goals while maintaining a manageable number for analysis: strains isolated over more than two decades, clones that collectively cause the vast majority of invasive infections in Israel [47], clones that are primarily associated with asymptomatic pharyngeal colonization [48], strains isolated from patients with a variety of clinical syndromes (bacteremia, skeletal system infection, or endocarditis) [42], and strains associated with clusters of disease in daycare center facilities [49]. Because the different genotyping schemes of *K*. *kingae* exhibit remarkable congruency [46], it was assumed that studying strains belonging to rare PFGE clones would increase the chances to detect novel capsule types. Thus, the strain collection was enriched with a large number of uncommon invasive as well as colonizing isolates.

### Ethics Statement

The Israeli isolates used in this study are part of a preexisting anonymized collection and as such did not require IRB approval for use.

### Molecular methods and strain manipulation

Targeted gene disruptions and complementation constructs in *K*. *kingae* were generated as previously described [4, 45]. Briefly, plasmid-based gene disruption constructs were created in *E*. *coli*, linearized, and introduced into *K*. *kingae* using natural transformation. Transformants were recovered by selectively plating on chocolate agar plates with the appropriate antibiotic. Gene disruptions and complementation constructs were confirmed by PCR.

The primers used in this study are listed in Table 7. To delete the capsule synthesis locus, we generated the plasmid pSwapEmpty. Briefly, fragments of homologous recombination targeting sequence corresponding to ~1 kb upstream of *csaA* and ~1 kb downstream of *csaA* were PCR amplified from strain KK01 genomic DNA using primers pSwapFor5’/pSwapRev5’ and pSwapFor3’/pSwapRev3’, respectively, and were ligated into pUC19. A kanamycin resistance cassette was then ligated into the pUC19 KpnI site, which is located between the cloned upstream and downstream homologous recombination targeting sequences, to generate pSwapEmpty. The plasmid was linearized with NdeI and transformed into strain KK01.

**Table 7:** Primers used in this study.

Primer name	Sequence 5’→3’
*hemB*For	CATTGGCGCAATCCGTCAGG
*arg*Rev	CTTGGGACGTTTGCTGTATC
*csa*ScreenFor	AAACGAGCCAAATTTTTTAC
*csa*ScreenRev	GCCTTATCTGTCATTATTCC
*csb*ScreenFor	CCTTGGCACGCAAAGCATAG
*csb*ScreenRev	AGCGCTTTATCTACCACCAC
*csc*ScreenFor	AACGAAAGTCATTCTAGATT
*csc*ScreenRev	CAGGGCTTCATTCCAAAGTG
*csd*ScreenFor	CATTGTTACCGATAATCAAATACC
*csd*ScreenRev	AGCGACTATATGCATGCTCC
pSwapFor5’	AGCTGAATTCCAGACGAAGACGACTACAAC
pSwapRev5’	AGCTGGTACCTTATTTAGTTTCCAACTTCGGAATC
pSwapFor3’	ACGTGTCGACTGCAGGCTGCTTTTTATTTATTTG
pSwapRev3’	ACGTAAGCTTGACGAACGCACCAAATTACGC
*csa*swapFor	ACGTGGTACCTTATCATAGAAAGCATTTCTTACTTTTGTATTATG
*csa*swapRev	ACGTGGATCCCTTTTTTGTTGAGTTTAAAGTGCAG
*csb*swapFor	ACGTGGTACCGATTTTTTGAGATGCAAATCAACGTC
*csb*swapRev	ACGTTCTAGACTTTTTGTTGAGTTTAAAGTGCAGG
*csc*swapFor	AAATAAGGTACCCGGGATATATAAGATGCGGATATTTTATATATTAATTTAATATC
*csc*swapRev	CGGTCGACTCTAGAGACCTTTGCAAAACTACCATG
*csd*swapFor	AAATAAGGTACCCGGGAACAACATATTCTGAAAATATCAATC
*csd*swapRev	CGGTCGACTCTAGAGGGGGCTATCCTAGATAAC
*lipA*screenFor	ATTGCCCTGCTTGCATTTCG
*lipA*screenRev	TCTTCCAACGCCACATACGG
*lipB*screenFor	ATACGCCGATTGGTTGCGAG
*lipB*screenRev	CCCGACAACGAAACGATAAG
*ctrABCD*screenFor	CCGTTATCCAACACCATAGC
*ctrABCD*screenRev	CCGTTATCCAACACCATAGC

To create the complementation/capsule swap constructs, the capsule synthesis loci were PCR amplified as follows: for the *csa* locus, using genomic DNA from strain KK01 and primers *csa*swapFor/*csa*swapRev; for the *csb* locus, using genomic DNA from strain PYKK58 and primers *csb*swapFor/*csb*swapRev; for the *csc* locus, using genomic DNA from strain PYKK060 and primers *csc*swapFor/*csc*swapRev; and for the *csd* locus, using genomic DNA from strain BB270 and primers *csd*swapFor/*csd*swapRev. The *csa* and *csb* locus amplicons were cloned into pSwapEmpty using standard restriction cloning, generating pSwap*csa* and pSwap*csb*, respectively. The *csc* and *csd* amplicons were cloned into pSwapEmpty using the Gibson Assembly Cloning kit (New England Biolabs, Ipswich, MA), generating pSwap*csc* and pSwap*csd*, respectively.

For the capsule swap studies, we transformed each swap construct (pSwap*csa*, pSwap*csb*, pSwap*csc*, or pSwap*csd*) harboring a kanamycin cassette into the nonencapsulated isogenic strain KK01Δ*csa* (Erm^R^) and screened for loss of Erm^R^ and gain of Kan^R^. For capsule synthesis locus complementation, the capsule swap plasmids pSwap*csa*, pSwap*csb*, pSwap*csc*, and pSwap*csd* were transformed into KK01Δ*csa*, PYKK58Δ*csb*, PYKK60Δ*csc*, and BB270Δ*csd*, respectively, using the unmarked transformation protocol described below.

To generate unmarked gene disruptions and complements, we used the following procedure without antibiotic selection. First, *K*. *kingae* was grown overnight on chocolate agar, resuspended in Brain Heart Infusion (BHI) broth containing 50 mM MgCl_2_ to an OD_600_ of 0.7, and diluted 1:25 in BHI broth. The initial dilution was then serially diluted 1:4 a total of 9 times, and 5 μl of each dilution was transferred to a microfuge tube containing 5 μl of linearized transforming plasmid at a concentration of 50 ng/μl. The 10 μl total mixture was then plated on chocolate plates and allowed to dry in ambient air conditions (approximately 5 minutes) before placement into the CO_2_ incubator at 37°C. Single colonies were screened by PCR for recombination at the locus of interest after two rounds of single colony purification of the potential transformants.

### Polysaccharide capsule extraction, purification, and analysis

In preparation for extraction and purification of capsule, the *pam* locus involved in synthesis of the galactan exopolysaccharide was deleted from the relevant strains [11]. Extraction, purification, and visualization of migration patterns on 7.5% SDS-PAGE gels using Alcian blue staining of capsule material were performed as previously described [4, 11].

#### Xylanase pretreatment

The purified samples were digested with endo-1,4-β-xylanase M1 from *Trichoderma viride* (Megazyme, Bray, Ireland). The digested samples were dialyzed using a 10-kDa regenerated cellulose membrane, and the retentates were freeze-dried.

#### De-O-acetylation

The sample solutions (~1.5 mg/0.5 ml) were adjusted to pH 11.0 by addition of 2 M ammonium hydroxide solution and incubated overnight at room temperature. The resulting solutions were dialyzed through a 3.5 kDa regenerated cellulose membrane to remove salts. The retentates were then deuterium-exchanged for NMR analysis.

#### NMR spectroscopy

The retentates after xylanase digestion (total amount) were deuterium exchanged 2 times by lyophilization in D_2_O. The dry residues were re-dissolved in 300 μl D_2_O (99.96%, Cambridge Isotopes) and placed in 3-mm OD NMR tubes. 1-D Proton and 2-D COSY, TOCSY, HSQC, HMBC and NOESY spectra were acquired at 55°C on an Agilent Inova 600 MHz spectrometer equipped with a cryoprobe using standard Agilent pulse sequences. Chemical shifts were measured relative to internal acetone peak (δ_H_/δ_C_ = 2.218/33.0 ppm).

#### Linkage analysis

For glycosyl linkage analysis, the samples were permethylated, depolymerized, reduced, and acetylated. The resultant partially methylated alditol acetates (PMAAs) were analyzed by gas chromatography-mass spectrometry (GC-MS) as described by Heiss et al. [50].

Approximately 1 mg quantities of the samples were used for linkage analysis. The samples were suspended in 200 μl of dimethyl sulfoxide and left to stir for 1 d. Permethylation was effected by two rounds of treatment with sodium hydroxide (15 min) and methyl iodide (45 min). Following sample workup, the permethylated material was hydrolyzed using 2 M TFA (2 h in sealed tube at 121°C), reduced with NaBD_4_, and acetylated using acetic anhydride/TFA. The resulting PMAAs were analyzed on an Agilent 7890A GC interfaced to a 5975C MSD (mass selective detector, electron impact ionization mode). Separation was performed on a 30 m Supelco SP-2331 bonded phase fused silica capillary column.

#### Linkage analysis of the Kdo residues

To prepare the partially methylated alditol acetate (PMAA) derivatives of Kdo, the permethylated polysaccharide samples were subjected to sequential steps: reduction of the Kdo carboxymethyl groups with lithium triethylborodeuteride (Superdeuteride, Aldrich, St. Louis, MO) in THF (200 μl, 2 h at room temperature); mild hydrolysis (0.1 M trifluoroacetic acid, 100°C, 30 min) to cleave the Kdo-ketosidic linkages; reduction of Kdo residues at C-2 carbonyl group (using NaBD_4_ in water/ethanol 1:1 v/v); normal hydrolysis (2 M trifluoroacetic acid, 100°C, 30 min) to cleave the sugar linkages; reduction of the newly formed aldehydo sugars (using NaBD_4_ in 50 mM NH_4_OH); and acetylation of the resulting partially methylated alditols to yield the PMAA derivatives. Acetylation was performed in acetonitrile-pyridine-acetic anhydride containing 4-*N*,*N′*-dimethylaminopyridine as a catalyst for 4 h at room temperature as described [51].

#### D and L determination

The samples were freeze-dried and hydrolyzed in 2M TFA (500 μl) at 120°C for 1.5 h. The hydrolysates were dissolved in 200 μl S-(+)-2-butanol (Fluka, St. Louis, MO), 15 μl acetyl chloride (Aldrich) was added, and nitrogen gas was bubbled through the solutions for 30 seconds. The mixtures were capped tightly and incubated at 80°C for 16 h. After incubation, the mixtures were dried under a stream of dry nitrogen and then redried with absolute methanol. A 250 μl volume of TMS reagent (Tri-Sil, Thermo Scientific Pierce, Waltham, MA) was added to the dry sample, and derivatization was carried out at 80°C for 20 min.

The same procedure was applied to authentic monosaccharide standards. Two sets of standards were derivatized, and either S-(+)-2-butanol or R-(-)-2-butanol was added separately to these standards. The derivatized samples and standards were analyzed on Agilent 5975C GC interfaced with 7890A MS detector.

#### Size exclusion chromatography (SEC)

The samples were dissolved in deionized water (2 ml), and 20 μl volumes of the resulting solutions were injected for separation on a Superdex-75 SEC column using an Agilent 1200 HPLC system. The eluent was 50 mM ammonium acetate, pH 5, the flow rate was 1 ml/min, and signal was monitored with an ELS detector. Peaks were integrated using Chemstation software.

#### Nanospray-mass spectrometry (NSI-MS)

NSI-MS analyses in full mass and MS_n_ mode were performed using an Orbitrap Fusion mass spectrometer (Thermo Fisher, Waltham, MA) equipped with a nanospray ion source. Intact capsule from type d strain BB270 was dissolved in 1 mM NaOH in 50% methanol and then infused directly into the instrument at a constant flow rate of 1 μl/ min. A full FTMS spectrum was collected at 30,000 resolution with 20 microscans. The capillary temperature was set at 210°C, and MS analysis was performed in the negative ion mode.

### Genetic screen of capsule synthesis loci

*K*. *kingae* sequence outside of the capsule synthesis locus from strain 269–492 was used to design flanking primers *hemB*For and *arg*Rev in the *hemB* (delta aminolevulinic-acid dehydratase) and *arg* (arginine-succinate synthase) genes flanking *csaA* (Table 7). PCR amplicons were sequenced, and the resulting sequence was the basis for design of interior primers specific for each of the four capsule synthesis loci. To screen for the presence of each locus, we used both universal flanking primers that amplified all capsule loci and locus-specific primers that annealed to the interior portion of each locus. The presence of either *csaA*, *csbABC*, *cscABC*, or *csdABC* (the *csa*, *csb*, *csc*, or *csd* capsule synthesis locus) was determined by PCR amplification using interior primers (see Table 7) and confirmed by determining the size and restriction map of the flanking primer amplicon.

### Restriction analysis

To obtain restriction digest patterns, PCR products were amplified using the primers *hemB*For and *arg*Rev listed in Table 7 in a total reaction volume of 25 μl. 2.5 μl of 10x digestion buffer and 1 μl of NruI enzyme (New England Biolabs) were mixed with PCR products and incubated overnight at 37°C. The digests were resolved on a 1.2% agarose gel and visualized for banding pattern.

### Statistical analysis

The statistical significance of the differences in the distribution of the different capsule types among carrier *vs*. invasive strains, among bacteremia *vs*. skeletal system infections, and among the PFGE clones was determined by the χ^2^ test using the Statistical Package for the Social Sciences (SPSS) version 21 software. The link between the different capsule types and invasiveness was further explored with a logistic regression model in which the capsule type with the lowest percentage of associated invasive strains was employed as a reference and the odds ratio (OR) for invasiveness, p-value, and 95% confidence intervals (CI) for the other capsule types were calculated. A p-value <0.05 was considered significant for all comparisons.

## Supporting Information

S1 Fig **GC-MS chromatograms of PMAAs in the linkage analysis of type b (A), type c (B), and type d (C) capsular polysaccharides.** The terminal residues (t-) arise from the non-reducing end of the polysaccharide; Kdo has two diastereomeric PMAAs due to the non-stereospecific reduction of C-2, which produces a new chiral center.
(TIF)

S2 Fig Mass spectra of the Kdo PMAAs detected in the linkage analysis. (A) Mass spectrum of the peak at 31.9 min in S1A Fig, demonstrating that type b CPS has 5-linked Kdo and (B) mass spectrum of the peak at 32.4 min in S2B Fig, demonstrating that type c has 4-linked Kdo.
(TIF)

S3 Fig NOESY NMR spectrum of the type c polysaccharide showing inter-residue correlations. (TIF)

S4 Fig NOESY NMR spectrum of the type d polysaccharide showing inter-residue correlations. (TIF)

S5 Fig 1D-^31^P-NMR spectrum of the type d polysaccharide showing peaks for phosphomono- (0.42 ppm) and diester (-1.20 ppm). (TIF)

S6 Fig **(A) SEC chromatogram and (B) negative ion NSI-MS of the type d polysaccharide, wherein n is the number of trisaccharide repeats.** This number is also equal to the charge state in the mass spectrum.
(TIF)

S7 Fig Analysis of non-encapsulated *K*. *kingae* isolates. Capsule type a strain KK01 (lane 1), capsule type b strain PYKK59 (lane 2), capsule type c strain PYKK60 (lane 3), capsule type d strain BB270 (lane 4), PYKK56 (lane 5), and PYKK183 (lane 6) were subjected to Alcian blue staining of surface extracts (A) and PCR of the capsule synthesis locus using the flanking primers (B), *csa-*specific primers (C), *csb*-specific primers (D), *csc-*specific primers (E), and *csd*-specific primers (F). PYKK56 and PYKK183 lack Alcian blue-stainable material in surface extracts. For PYKK56, the capsule synthesis locus flanking PCR amplicon is smaller than all of the four control amplicons, and the *csa*-specific PCR amplicon is smaller than the control *csa-*specific PCR amplicon. Sequencing of the capsule synthesis locus flanking PCR amplicon from PYKK56 (Panel B, Lane 5) revealed a *csaA* gene with a 512 bp deletion in the open reading frame (G), which also introduces a frameshift mutation leading to a predicted truncated CsaA protein of 316 amino acids versus the wild type 811 amino acid protein. The capsule locus flanking and capsule locus specific PCRs failed to generate amplicons for PYKK183.
(TIF)

S1 Table Distribution of capsular types among invasive and asymptomatically carried *K*. *kingae* strains. (PDF)

S2 Table Distribution of capsular types by invasive *K*. *kingae* disease. (PDF)

S3 Table Distribution of capsule types among the 16 most common *K*. *kingae* PFGE clones. (PDF)

S4 Table Israeli strain collection used in this study. (PDF)
