# Supplementary figures and images for: Kingella kingae Expresses Four Structurally Distinct Polysaccharide Capsules That Differ in Their Correlation with Invasive Disease
Source: PLoS Pathog. 2016 Oct 19;12(10):e1005944. doi: 10.1371/journal.ppat.1005944 (PMC5070880; doi:10.1371/journal.ppat.1005944)

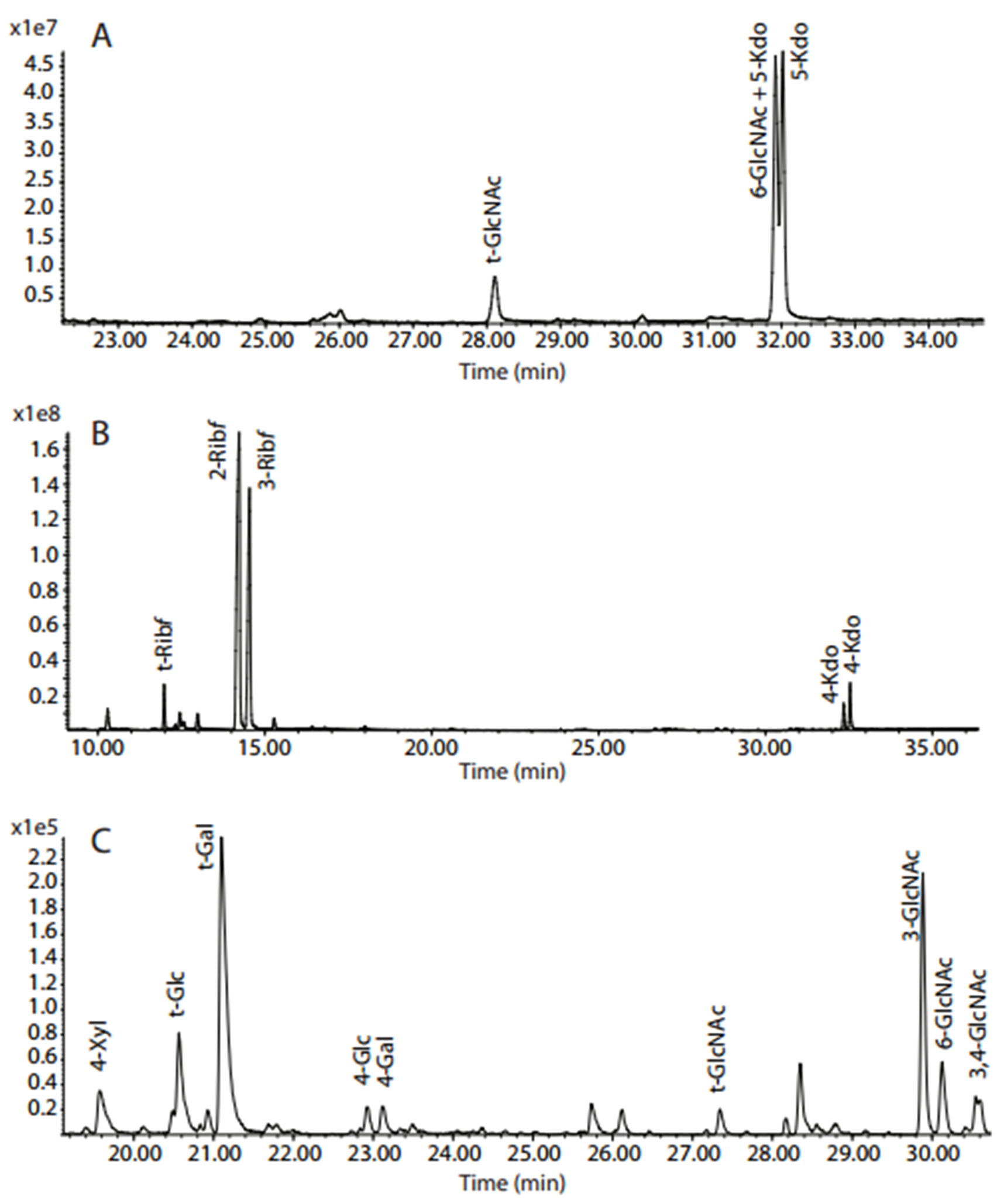

Supplement: S1 Fig — GC-MS chromatograms of PMAAs in the linkage analysis of type b (A), type c (B), and type d (C) capsular polysaccharides. The terminal residues (t-) arise from the non-reducing end of the polysaccharide; Kdo has two diastereomeric PMAAs due to the non-stereospecific reduction of C-2, which produces a new chiral center. (TIF) [file ppat.1005944.s001.tif]

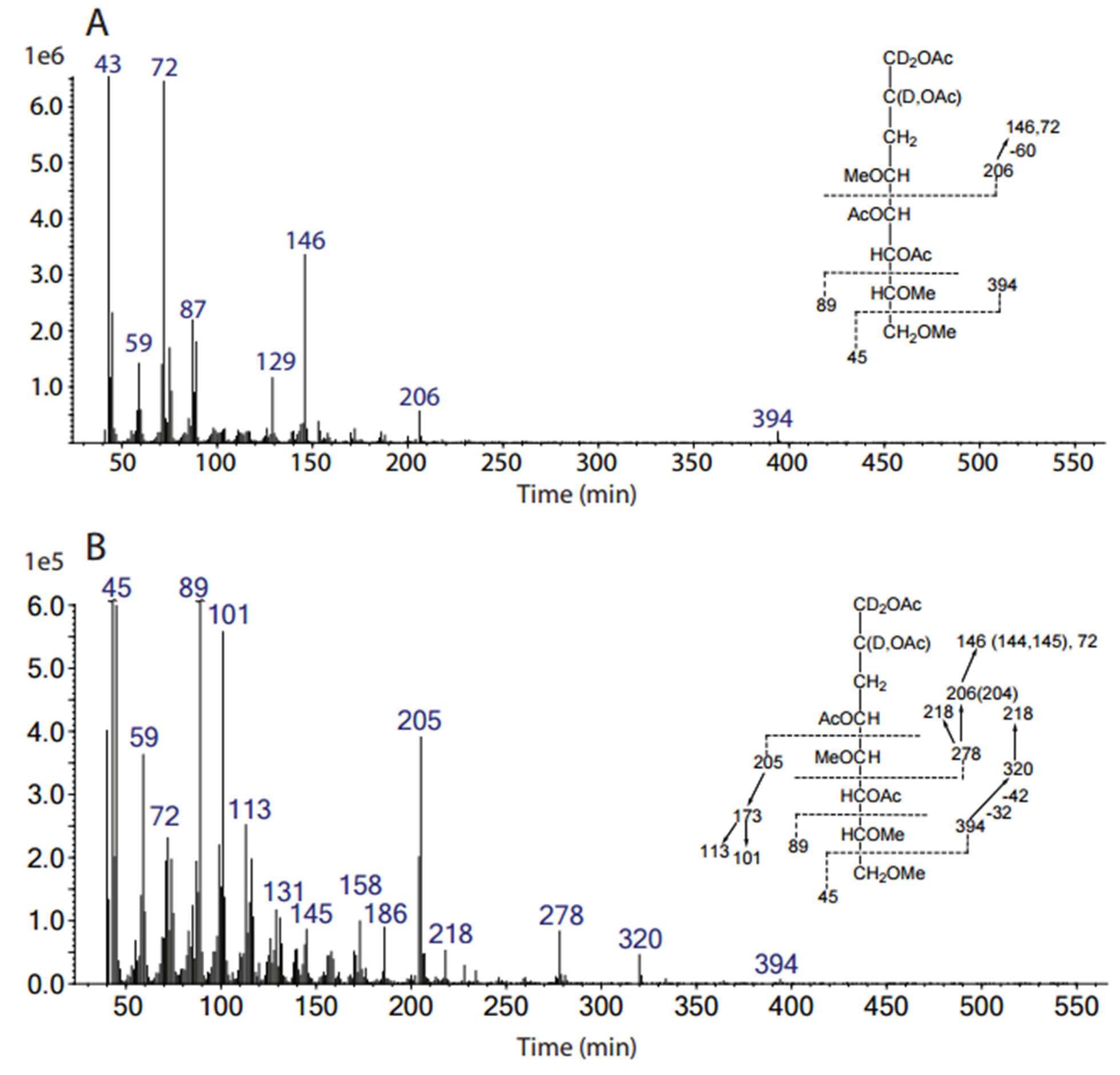

Supplement: S2 Fig — (A) Mass spectrum of the peak at 31.9 min in S1A Fig, demonstrating that type b CPS has 5-linked Kdo and (B) mass spectrum of the peak at 32.4 min in S2B Fig, demonstrating that type c has 4-linked Kdo. (TIF) [file ppat.1005944.s002.tif]

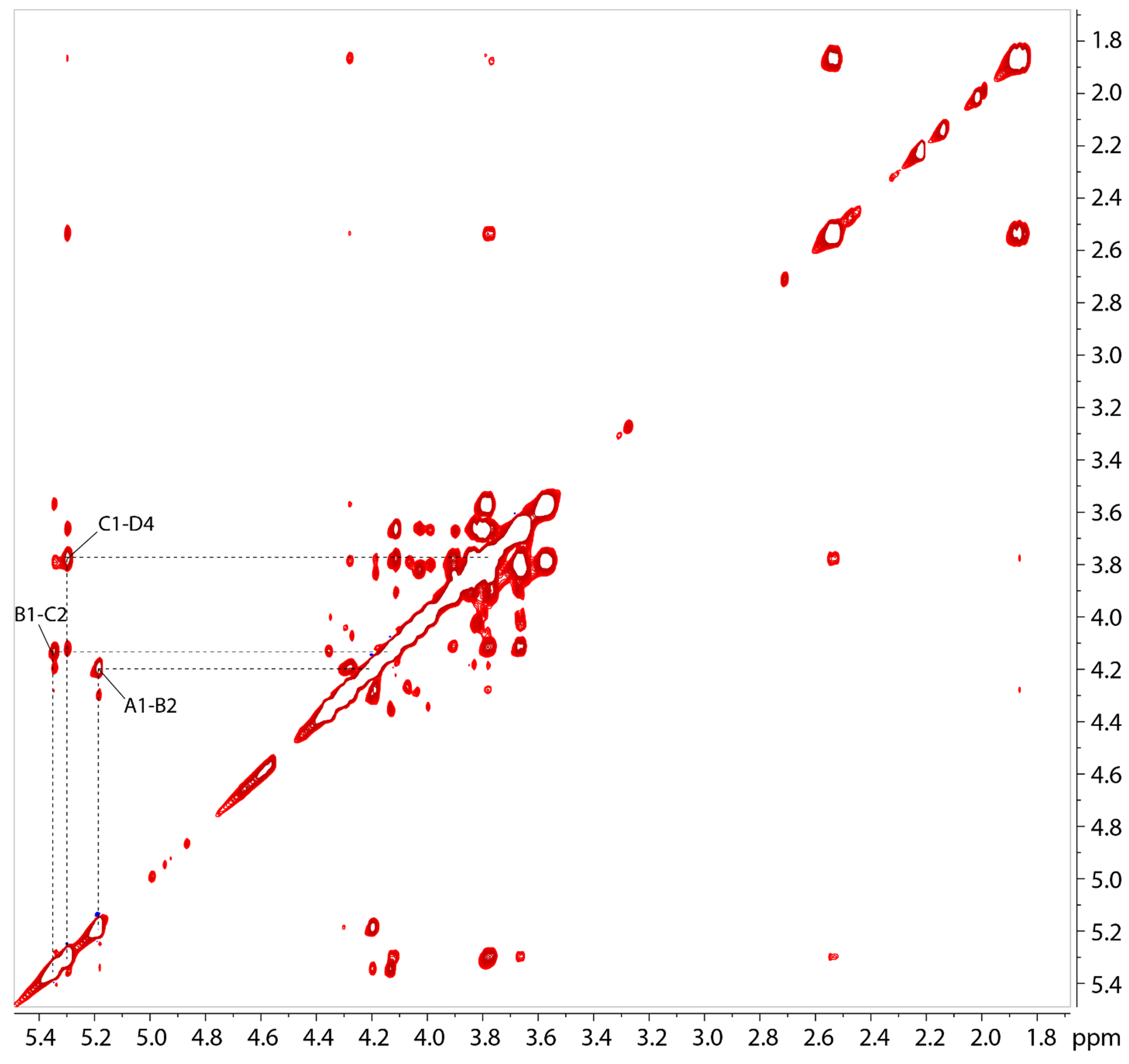

Supplement: S3 Fig — (TIF) [file ppat.1005944.s003.tif]

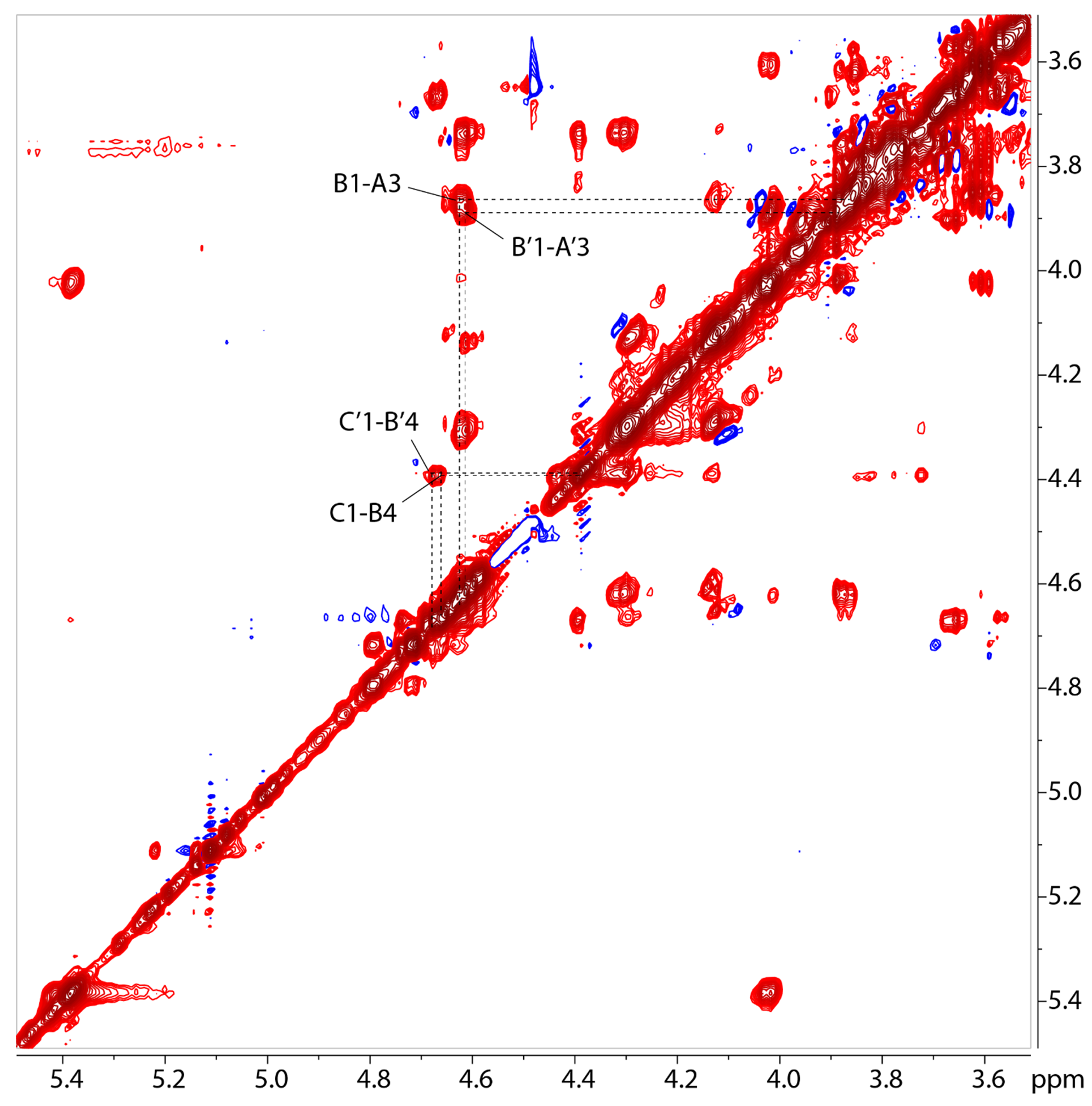

Supplement: S4 Fig — (TIF) [file ppat.1005944.s004.tif]

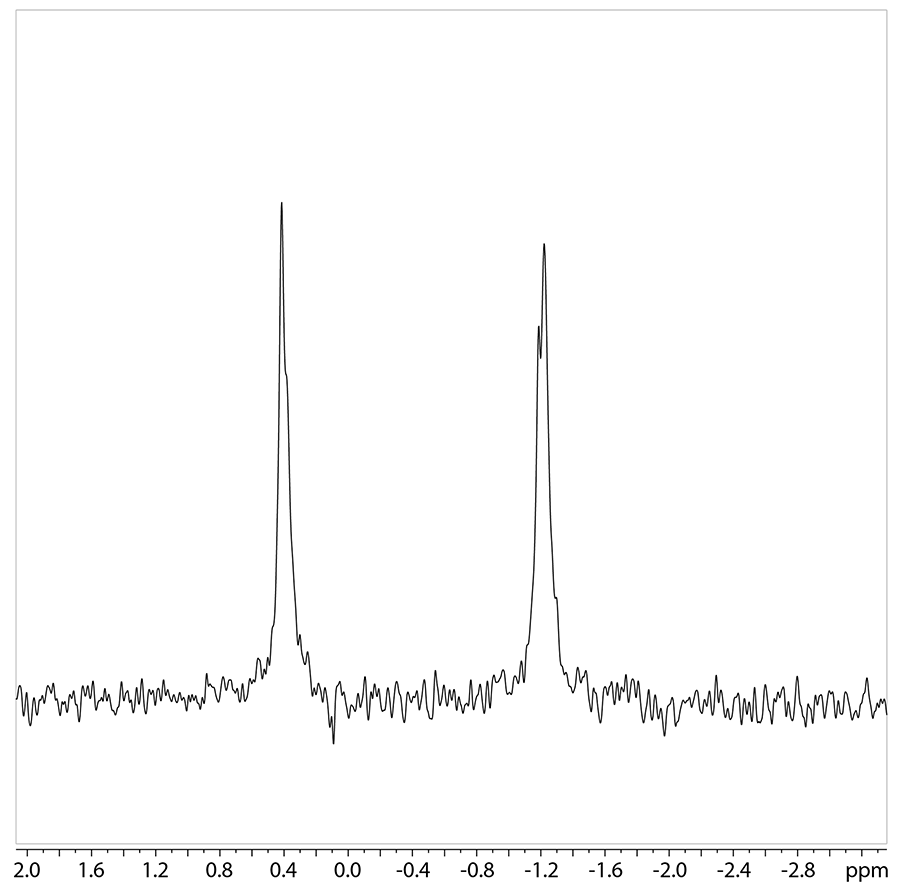

Supplement: S5 Fig — (TIF) [file ppat.1005944.s005.tif]

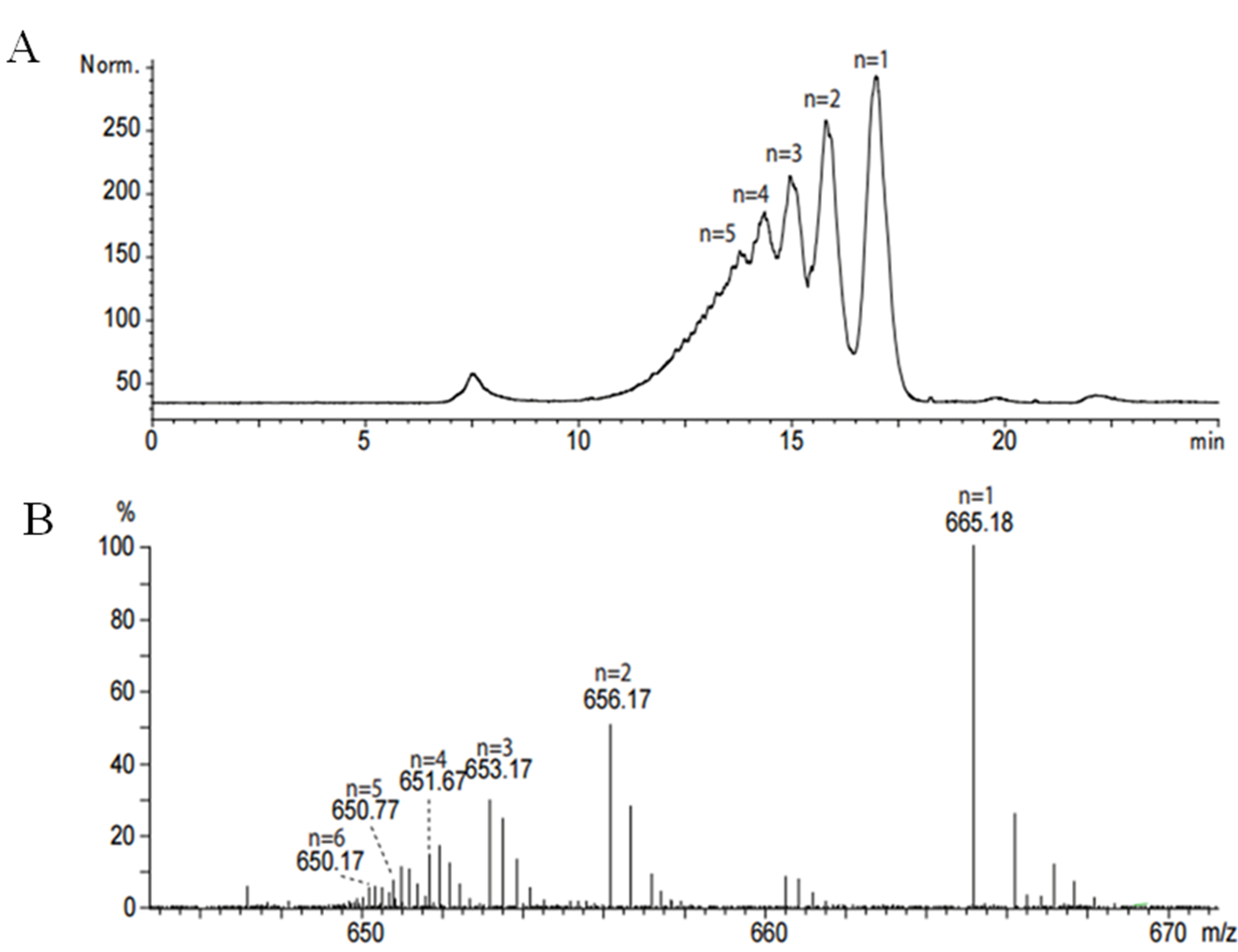

Supplement: S6 Fig — (A) SEC chromatogram and (B) negative ion NSI-MS of the type d polysaccharide, wherein n is the number of trisaccharide repeats. This number is also equal to the charge state in the mass spectrum. (TIF) [file ppat.1005944.s006.tif]

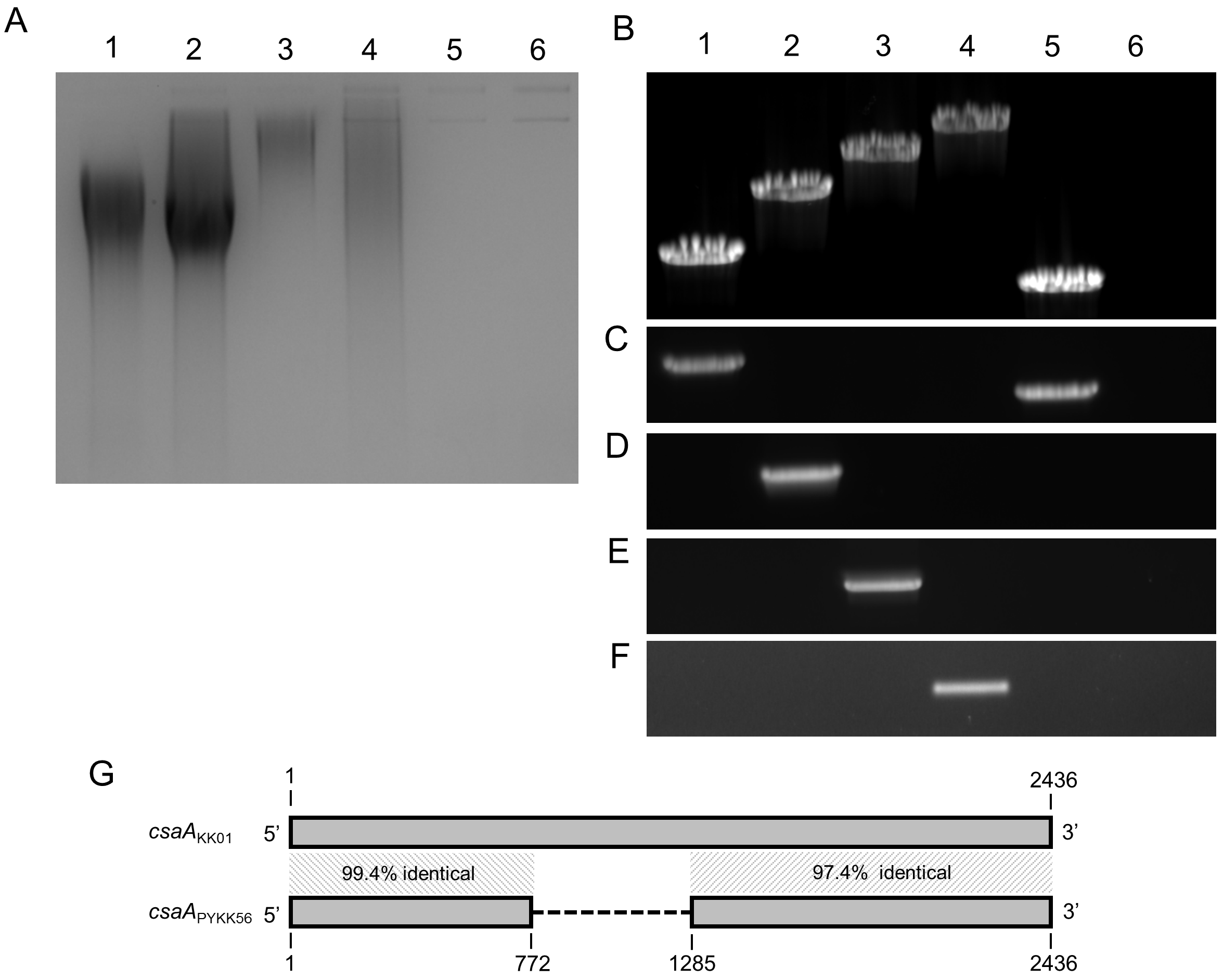

Supplement: S7 Fig — Capsule type a strain KK01 (lane 1), capsule type b strain PYKK59 (lane 2), capsule type c strain PYKK60 (lane 3), capsule type d strain BB270 (lane 4), PYKK56 (lane 5), and PYKK183 (lane 6) were subjected to Alcian blue staining of surface extracts (A) and PCR of the capsule synthesis locus using the flanking primers (B), csa-specific primers (C), csb-specific primers (D), csc-specific primers (E), and csd-specific primers (F). PYKK56 and PYKK183 lack Alcian blue-stainable material in surface extracts. For PYKK56, the capsule synthesis locus flanking PCR amplicon is smaller than all of the four control amplicons, and the csa-specific PCR amplicon is smaller than the control csa-specific PCR amplicon. Sequencing of the capsule synthesis locus flanking PCR amplicon from PYKK56 (Panel B, Lane 5) revealed a csaA gene with a 512 bp deletion in the open reading frame (G), which also introduces a frameshift mutation leading to a predicted truncated CsaA protein of 316 amino acids versus the wild type 811 amino acid protein. The capsule locus flanking and capsule locus specific PCRs failed to generate amplicons for PYKK183. (TIF) [file ppat.1005944.s007.tif]
