# Supplementary material for: Kingella kingae Expresses Four Structurally Distinct Polysaccharide Capsules That Differ in Their Correlation with Invasive Disease
Source: PLoS Pathog. 2016 Oct 19;12(10):e1005944. doi: 10.1371/journal.ppat.1005944 (PMC5070880; doi:10.1371/journal.ppat.1005944)
Supplement: S1 Table — (PDF) [file ppat.1005944.s008.pdf]

S1 Table. Distribution of capsule types among invasive and asymptomatically carried *K. kingae* strains.

Clinical condition	n	Capsule type				
		a	b	c	d	none
Carriage	239	117 (49.0%)	46 (19.2%)	29 (12.1%)	47 (19.7%)	0 (0.0%)
Invasive	178	80 (44.9%)	91 (51.1%)	4 (2.2%)	2 (1.7%)	1 (0.6%)
Total	417	197 (47.2%)	137 (32.9%)	33 (7.9%)	49 (11.8%)	1 (0.2%)
