# Supplementary material for: Kingella kingae Expresses Four Structurally Distinct Polysaccharide Capsules That Differ in Their Correlation with Invasive Disease
Source: PLoS Pathog. 2016 Oct 19;12(10):e1005944. doi: 10.1371/journal.ppat.1005944 (PMC5070880; doi:10.1371/journal.ppat.1005944)
Supplement: S2 Table — (PDF) [file ppat.1005944.s009.pdf]

S2 Table. Distribution of capsular types by invasive *K. kingae* disease.

Invasive disease	n	Capsule type				
		**a**	**b**	**c**	**d**	**none**
Bacteremia/ Bacteremic LTB[a]	71 (39.8%)	26 (37.7%)	45 (63.3%)	0 (0.0%)	0 (0.0%)	0 (0.0%)
Endocarditis	11 (6.2%)	6 (54.5%)	3 (27.3%)	2 (18.2%)	0 (0.0%)	0 (0.0%)
Skeletal[b]	96 (53.9%)	48 (50.0%)	43 (44.8%)	2 (2.1%)	2 (2.1%)	1 (1.0%)
Total	178 (100%)	80 (44.9%)	91 (51.1%)	4 (2.2%)	2 (1.1%)	1 (0.6%)

[a]Bacteremia includes 2 cases of bacteremia/LTB. LTB: laryngotracheobronchitis.

[b]Skeletal includes cases of abortive osteoarthritis, septic arthritis, osteomyelitis, and tenosynovitis.
