# Supplementary material for: Kingella kingae Expresses Four Structurally Distinct Polysaccharide Capsules That Differ in Their Correlation with Invasive Disease
Source: PLoS Pathog. 2016 Oct 19;12(10):e1005944. doi: 10.1371/journal.ppat.1005944 (PMC5070880; doi:10.1371/journal.ppat.1005944)
Supplement: S3 Table — (PDF) [file ppat.1005944.s010.pdf]

S3 Table. Distribution of capsule types among the 16 most common *K. kingae* PFGE clones.

PFGE clone	n	Capsule type			
		a	b	c	d
A	22	22	0	0	0
B	34	32	2	0	0
C	20	20	0	0	0
D	23	0	0	23	0
F	13	0	2	0	11
G	21	2	1	0	18
H	37	35	2	0	0
J	17	17	0	0	0
K	78	4	74	0	0
M	7	6	1	0	0
N	21	1	20	0	0
P	7	7	0	0	0
R	9	1	0	8	0
S	14	14	0	0	0
U	12	0	0	0	12
V	8	0	8	0	0
