# Supplementary material for: Kingella kingae Expresses Four Structurally Distinct Polysaccharide Capsules That Differ in Their Correlation with Invasive Disease
Source: PLoS Pathog. 2016 Oct 19;12(10):e1005944. doi: 10.1371/journal.ppat.1005944 (PMC5070880; doi:10.1371/journal.ppat.1005944)
Supplement: S4 Table — (PDF) [file ppat.1005944.s011.pdf]

S4 Table. Israeli strain collection used in this study.

Isolate	Date	Syndrome	PFGE clone	Capsule type
AA393	2006	Carrier	A	a
AA478	2007	Carrier	A	a
BB114	2006	Carrier	A	a
BB149	2006	Carrier	A	a
BB217	2007	Carrier	A	a
BB280	2007	Carrier	A	a
BB284	2006	Carrier	A	a
CC141	2007	Carrier	A	a
CC222	2007	Carrier	A	a
CC410	2006	Carrier	A	a
D2396	2006	Carrier	A	a
EE160	2006	Carrier	A	a
EE515	2007	Carrier	A	a
EE672	2007	Carrier	A	a
EPA153	2007	Carrier	A	a
PVC1580	2006	Carrier	A	a
PVC1588	2006	Carrier	A	a
PVC1619	2006	Carrier	A	a
PVC1629	2006	Carrier	A	a
PVC1637	2006	Carrier	A	a
KK245	2005	Bacteremia	A	a
KK247	2006	Endocarditis	A	a
AA090	2007	Carrier	B	a
AA242	2006	Carrier	B	a
AA503	2007	Carrier	B	a
AA574	2007	Carrier	B	a
AA644	2007	Carrier	B	a
BB012	2006	Carrier	B	a
BB711	2008	Carrier	B	a
CC318	2007	Carrier	B	a
CC441	2008	Carrier	B	a
CC546b	2007	Carrier	B	a
D2332	2009	Carrier	B	a
KK176	2002	Bacteremia	B	a
KK186	2002	Bacteremia	B	a
KK217	2004	Bacteremia	B	a
KK422	2010	Bacteremia	B	a
KK424	2010	Bacteremia	B	a
KK61	1995	Bacteremia	B	b
KK98	1992	Bacteremia	B	a
KK103	1992	Septic arthritis	B	a
KK123	1997	Septic arthritis	B	a
KK142	1998	Septic arthritis	B	a
KK167	2000	Septic arthritis	B	a

Isolate	Date	Syndrome	PFGE clone	Capsule type
KK275	2008	Septic arthritis	B	a
KK277	2009	Septic arthritis	B	a
KK402	2007	Septic arthritis	B	a
KK425	2011	Septic arthritis	B	a
KK427	2012	Septic arthritis	B	a
KK429	2011	Septic arthritis	B	a
KK81	1991	Septic arthritis	B	a
Ra12	2003	Septic arthritis	B	b
KK157	1999	Tenosynovitis	B	a
KK256	2007	Tenosynovitis	B	a
KK409	2010	Endocarditis	B	a
AA080	2006	Carrier	C	a
AA392	2006	Carrier	C	a
AA532	2007	Carrier	C	a
AA574	2007	Carrier	C	a
BB099	2006	Carrier	C	a
BB230	2007	Carrier	C	a
BB368	2007	Carrier	C	a
BB587	2007	Carrier	C	a
CC047	2006	Carrier	C	a
CC455	2006	Carrier	C	a
CC500a	2007	Carrier	C	a
CC505	2007	Carrier	C	a
CC623b	2007	Carrier	C	a
D2116	2007	Carrier	C	a
EPA 011	2007	Carrier	C	a
KK113	1996	Carrier	C	a
PVC1590	2006	Carrier	C	a
PVC1622	2006	Carrier	C	a
PVC1714	2007	Carrier	C	a
PYKK125	1996	Carrier	C	a
KK438	2013	Septic arthritis	cc	b
AA097	2006	Carrier	D	c
AA392	2008	Carrier	D	c
AA478	2006	Carrier	D	c
AA491	2006	Carrier	D	c
AA545	2007	Carrier	D	c
BB060	2006	Carrier	D	c
BB151	2007	Carrier	D	c
BB371	2007	Carrier	D	c
BB477	2007	Carrier	D	c
BB575	2007	Carrier	D	c
BB653	2006	Carrier	D	c
CC079	2006	Carrier	D	c
CC152	2007	Carrier	D	c

Isolate	Date	Syndrome	PFGE clone	Capsule type
CC171	2007	Carrier	D	c
CC499a	2007	Carrier	D	c
D2464	2006	Carrier	D	c
D7006	2007	Carrier	D	c
PVC1595	2006	Carrier	D	c
PVC1681	2007	Carrier	D	c
Sch194	2010	Carrier	D	c
KK185	2002	Septic arthritis	D	c
KK88	1996	Septic arthritis	D	c
KK60	1994	Endocarditis	D	c
AA207	2006	Carrier	E	b
D2312	2006	Carrier	E	b
KK131	1997	Bacteremia	E	b
KK147	1998	Bacteremia	E	b
AA046	2006	Carrier	F	d
AA139	2006	Carrier	F	d
AA528a	2007	Carrier	F	d
AA545	2008	Carrier	F	d
BB033	2006	Carrier	F	b
CC388	2006	Carrier	F	b
CC397	2007	Carrier	F	d
D2373	2006	Carrier	F	d
EE200	2006	Carrier	F	d
EE299	2006	Carrier	F	d
PVC1618	2006	Carrier	F	d
PVE3339	2007	Carrier	F	d
Sch187	2010	Carrier	F	d
AA481	2007	Carrier	G	d
BB012	2007	Carrier	G	a
BB181	2007	Carrier	G	d
BB237	2007	Carrier	G	d
BB655b	2007	Carrier	G	d
CC220	2008	Carrier	G	a
CC380	2006	Carrier	G	d
D7453	2006	Carrier	G	d
D7674	2007	Carrier	G	d
KK16	1994	Carrier	G	d
KK18	1994	Carrier	G	d
Kk20	1997	Carrier	G	d
Kk24	1994	Carrier	G	d
Kk25	1998	Carrier	G	d
KK3	1994	Carrier	G	d
KK52	1994	Carrier	G	d
KK7	1994	Carrier	G	d
KK9	1994	Carrier	G	b

Isolate	Date	Syndrome	PFGE clone	Capsule type
PVC1587	2006	Carrier	G	d
PVW4012	2006	Carrier	G	d
Vir5453	2006	Carrier	G	d
AA528a	2008	Carrier	gg	a
CC516	2008	Carrier	gg	a
D2363	2008	Carrier	gg	a
AA026	2006	Carrier	H	a
AA469b	2007	Carrier	H	b
AA622	2007	Carrier	H	a
BB114	2008	Carrier	H	a
BB300	2007	Carrier	H	a
CC047	2007	Carrier	H	a
CC500a	2008	Carrier	H	a
CC505	2007	Carrier	H	a
KK12	1994	Carrier	H	a
PVC1572	2006	Carrier	H	a
PYP8oropharynx	2005	Carrier	H	b
KK126	1997	Bacteremia	H	a
KK129	1997	Bacteremia	H	a
KK130	1997	Bacteremia	H	a
KK136	1998	Bacteremia	H	a
KK148	1999	Bacteremia	H	a
KK154	1999	Bacteremia	H	a
KK193	2003	Bacteremia	H	a
KK221	2004	Bacteremia	H	a
KK276	2009	Bacteremia	H	a
KK407	2011	Bacteremia	H	a
B11058	2005	Septic arthritis	H	a
B1878	2006	Septic arthritis	H	a
B392	2006	Septic arthritis	H	a
B5418	2007	Septic arthritis	H	a
B8038	2008	Septic arthritis	H	a
B8693	2008	Septic arthritis	H	a
KahiriBlood	2007	Septic arthritis	H	a
KK127	1997	Septic arthritis	H	a
KK179	2002	Septic arthritis	H	a
KK206	2004	Septic arthritis	H	a
KK413	2010	Septic arthritis	H	a
KK64	1990s	Septic arthritis	H	a
KK82	1990	Septic arthritis	H	a
KK83	1991	Septic arthritis	H	a
KK70	1993	Osteomyelitis	H	a
KK264	2008	Abortive OA	H	a
AA139f	2009	Carrier	I	a
AA393	2007	Carrier	I	a

Isolate	Date	Syndrome	PFGE clone	Capsule type
CC013	2006	Carrier	I	b
CC318	2008	Carrier	I	a
PYKK200	2004	Carrier	I	a
AA001	2006	Carrier	J	a
AA010	2006	Carrier	J	a
AA105	2006	Carrier	J	a
AA255	2006	Carrier	J	a
AA265	2006	Carrier	J	a
BB040	2006	Carrier	J	a
BB632a	2007	Carrier	J	a
CC218	2007	Carrier	J	a
CC223	2006	Carrier	J	a
CC660	2007	Carrier	J	a
D2166a	2008	Carrier	J	a
D2508	2007	Carrier	J	a
D7182	2007	Carrier	J	a
D7517	2008	Carrier	J	a
Sch2407	2013	Carrier	J	a
B10615	2008	Septic arthritis	J	a
B9853	2006	Septic arthritis	J	a
AA242(B)	2006	Carrier	K	b
AA261a	2007	Carrier	K	b
AA351	2008	Carrier	K	b
AA417	2007	Carrier	K	b
AA469	2006	Carrier	K	a
BB631	2008	Carrier	K	b
BB711	2007	Carrier	K	b
BB711	2007	Carrier	K	b
CC141	2007	Carrier	K	a
CC220	2007	Carrier	K	b
CC223	2007	Carrier	K	b
CC731a	2007	Carrier	K	b
D2030	2007	Carrier	K	b
D2166	2006	Carrier	K	b
KK1	1994	Carrier	K	b
KK10	1998	Carrier	K	b
KK106	1994	Carrier	K	b
KK107	1996	Carrier	K	b
KK2	1994	Carrier	K	b
KK203	2004	Carrier	K	b
KK28	1994	Carrier	K	b
KK33	1998	Carrier	K	b
KK35	1994	Carrier	K	b
KK36	1994	Carrier	K	b
KK37	1994	Carrier	K	b

Isolate	Date	Syndrome	PFGE clone	Capsule type
KK39	1998	Carrier	K	b
KK4	1994	Carrier	K	b
KK45	1994	Carrier	K	b
KK6	1994	Carrier	K	b
KK86	1990s	Carrier	K	b
PYKK02	1994	Carrier	K	b
B087192	2012	Bacteremia	K	b
KK109	1996	Bacteremia	K	b
KK118	1996	Bacteremia	K	b
KK121	1996	Bacteremia	K	b
KK132	1997	Bacteremia	K	b
KK134	1997	Bacteremia	K	b
KK139	1998	Bacteremia	K	b
KK151	1999	Bacteremia	K	b
KK159	2000	Bacteremia	K	b
KK163	2000	Bacteremia	K	b
KK173	2001	Bacteremia	K	b
KK175	2002	Bacteremia	K	b
KK178	2002	Bacteremia	K	b
KK198	2004	Bacteremia	K	b
KK219	2004	Bacteremia	K	b
KK222	2005	Bacteremia	K	b
KK246	2006	Bacteremia	K	b
KK265	2008	Bacteremia	K	b
KK403	2008	Bacteremia	K	b
KK404	2008	Bacteremia	K	b
KK423	2010	Bacteremia	K	b
KK432	2012	Bacteremia	K	b
KK71	1994	Bacteremia	K	b
KK89	1995	Bacteremia	K	b
KK92	1996	Bacteremia	K	b
Ra1854	2005	Bacteremia	K	b
ItzhakBlood	2007	Bacteremia/LTB	K	b
KK266	2008	Bacteremia/LTB	K	b
KK102	1992	Septic arthritis	K	b
KK145	1998	Septic arthritis	K	b
KK150	1999	Septic arthritis	K	b
KK156	1999	Septic arthritis	K	b
KK166	2000	Septic arthritis	K	b
KK170	2001	Septic arthritis	K	b
KK177	2002	Septic arthritis	K	b
KK274	2009	Septic arthritis	K	b
KK400	2009	Septic arthritis	K	b
KK401	2009	Septic arthritis	K	b
KK415	2011	Septic arthritis	K	b

Isolate	Date	Syndrome	PFGE clone	Capsule type
KK418	2011	Septic arthritis	K	b
KK426	2011	Septic arthritis	K	b
KK428	2011	Septic arthritis	K	b
KK80	1996	Septic arthritis	K	b
KK95	1997	Septic arthritis	K	b
Ra277	2003	Septic arthritis	K	a
KK101	1992	Osteomyelitis	K	b
KK182	2002	Abortive OA	K	b
KK79	1994	Abortive OA	K	a
KK199	2004	Endocarditis	K	b
KK411	2008	Endocarditis	K	b
AA261	2006	Carrier	M	a
AA622	2006	Carrier	M	a
CC731a	2009	Carrier	M	a
D7517	2007	Carrier	M	a
Sch559	2011	Carrier	M	b
9001970	2012	Septic arthritis	M	a
KK416	2011	Endocarditis	M	a
AA068	2007	Carrier	N	b
KK146	1998	Bacteremia	N	b
KK250	2007	Bacteremia	N	b
KK59	1993	Bacteremia	N	b
KK96	1992	Bacteremia	N	b
BB11960	2004	Septic arthritis	N	b
KK135	1998	Septic arthritis	N	b
KK140	1998	Septic arthritis	N	b
KK155	1999	Septic arthritis	N	b
KK162	2000	Septic arthritis	N	b
KK189	2002	Septic arthritis	N	b
KK57	1995	Septic arthritis	N	b
KK58	1993	Septic arthritis	N	b
KK72	1991	Septic arthritis	N	b
KK75	1991	Septic arthritis	N	b
KK84	1990s	Septic arthritis	N	a
KK94	1995	Septic arthritis	N	b
KK141	1998	Osteomyelitis	N	b
KK74	1991	Tenosynovitis	N	b
KK158	1999	Abortive OA	N	b
KK180	2002	Endocarditis	N	b
BB016	2006	Carrier	O	a
KK97	1992	Bacteremia	O	a
B4283	2000s	Septic arthritis	O	a
KK138	1998	Septic arthritis	O	a
KK164	2000	Bacteremia	P	a
KK271	2009	Bacteremia	P	a

Isolate	Date	Syndrome	PFGE clone	Capsule type
KK93	1995	Bacteremia	P	a
KK144	1998	Septic arthritis	P	a
KK128	1997	Endocarditis	P	a
KK190	2002	Endocarditis	P	a
KK197	2003	Endocarditis	P	a
KK445	2013	Septic arthritis	pp	a
BB463	2007	Carrier	Q	a
KK114	1996	Carrier	Q	a
KK406	2010	Bacteremia	Q	a
KK253	2007	Septic arthritis	Q	a
AA038	2006	Carrier	R	a
CC062	2007	Carrier	R	c
CC684	2007	Carrier	R	c
CC762	2006	Carrier	R	c
D2312	2007	Carrier	R	c
D2363	2007	Carrier	R	c
D2523	2007	Carrier	R	c
D7592	2007	Carrier	R	c
D7674	2007	Carrier	R	c
AA315	2006	Carrier	S	a
BB300	2006	Carrier	S	a
BB572	2007	Carrier	S	a
BB587	2007	Carrier	S	a
CC093a	2008	Carrier	S	a
D2593	2007	Carrier	S	a
EPA326	2007	Carrier	S	a
PV1748	2007	Carrier	S	a
PVC1639	2006	Carrier	S	a
PVE3506	2007	Carrier	S	a
KK152	1999	Bacteremia	S	a
KK243	2005	Bacteremia	S	a
KK249	2006	Bacteremia	S	a
KK56	1994	Septic arthritis	S	a*
AA174	2006	Carrier	U	d
AA392	2007	Carrier	U	d
AA574	2006	Carrier	U	d
BB270	2007	Carrier	U	d
CC441	2006	Carrier	U	d
EE299	2006	Carrier	U	d
KK14	1993	Carrier	U	d
KK19	1997	Carrier	U	d
KK29	1998	Carrier	U	d
KK53	1998	Carrier	U	d
PVC1746	2006	Carrier	U	d
9010090	2011	Septic arthritis	U	d

Isolate	Date	Syndrome	PFGE clone	Capsule type
BB285	2006	Carrier	V	b
BB307	2007	Carrier	V	b
CC254	2006	Carrier	V	b
KK181	2002	Bacteremia	V	b
KK242	2005	Bacteremia	V	b
KK430	2012	Bacteremia	V	b
KK143	1998	Septic arthritis	V	b
KK405	2010	Septic arthritis	V	b
BB631	2006	Carrier	W	d
KK171	2001	Septic arthritis	W	d
AA351	2006	Carrier	X	a
BB033	2006	Carrier	Y	d
KK449	2014	Bacteremia	Y	b
D2030	2007	Carrier	yy	a
KK223	2005	Osteomyelitis	Tna	b
KK225	2005	Carrier	Tnb	a
KK183	2002	Septic arthritis	Tnc	none*
KK421	2010	Septic arthritis	Tnc	a
KK133	1997	Bacteremia	ua	b
B8784	2005	Septic arthritis	ua	b
KK240	2005	Septic arthritis	ua	b
KK168	2001	Septic arthritis	ub	a
KK410	2008	Septic arthritis	uc	b
KK419	2011	Septic arthritis	uc	b
KK165	2000	Septic arthritis	ue	a
KK220	2004	Septic arthritis	uf	b
KK244	2005	Bacteremia	ug	b
KK153	1999	Endocarditis	uj	a
KK172	2001	Bacteremia	ul	b
KK184	2002	Septic arthritis	um	a
BB270	2007	Carrier	unique 10	b
AA265	2007	Carrier	unique 11	d
Sch2540	2012	Carrier	unique 2	c
BB149	2007	Carrier	unique 3	a
BB575	2007	Carrier	unique 4	a
D2312	2006	Carrier	unique 6	a
Sch2981	2013	Carrier	unique 7	a
KK440	2013	Bacteremia	unique 8	b
KK263	2008	Bacteremia	ur	b
KK417	2011	Septic arthritis	us	a
KK420	2011	Osteomyelitis	ut	a
KK433	2012	Septic arthritis	uu	b
KK431	2012	Bacteremia	uw	b
KK434	2013	Septic arthritis	uz	b
CC516	2007	Carrier	α	d

Isolate	Date	Syndrome	PFGE clone	Capsule type
D7676	2007	Carrier	α	d
Sch462	2011	Carrier	δ	a
Sch87	2010	Carrier	δ	a
Sch1614	2012	Carrier	ϑ	b
CC623a	2007	Carrier	γ	d
ItzhakPH2	2007	Carrier	π	a
KK408	2010	Bacteremia	π	a
Sch429	2011	Carrier	σ	a
BB307	2007	Carrier	ψ	d
AA393c	2007	Carrier	not typed	a
AA644g	2007	Carrier	not typed	a
BB655	2006	Carrier	not typed	a
AA469	2007	Carrier	not typed	b

*Strains KK56 and KK183 do not have surface capsule based on Alcian blue staining of surface extracts (Fig S7A).
